# Ru Cluster Incorporated NiMoO(P)_4_ Nanosheet Arrays as High‐Efficient Bifunctional Catalyst for Wind/Solar‐To‐Hydrogen Generation Systems

**DOI:** 10.1002/advs.202304179

**Published:** 2023-10-25

**Authors:** Shengye Wu, Ding Chen, Shang Li, Yuting Zeng, Tao Wang, Jian Zhang, Jun Yu, Shichun Mu, Haolin Tang

**Affiliations:** ^1^ State Key Laboratory of Advanced Technology for Materials Synthesis and Processing Wuhan University of Technology Wuhan 430070 China; ^2^ Key Laboratory of Fuel Cell Technology of Hubei Province Wuhan University of Technology Wuhan 430070 China

**Keywords:** bifunctional catalysts, hydrogen production, solar/wind‐to‐hydrogen integrated system, water splitting

## Abstract

Developing cost‐efficient bifunctional water splitting catalysts is crucial for sustainable hydrogen energy applications. Herein, ruthenium (Ru)‐incorporated and phosphorus (P)‐doped nickel molybdate (Ru‐NiMoO(P)_4_) nanosheet array catalysts are synthesized. Due to the synergy of Ru clusters and NiMoO(P)_4_ by the modulated electronic structure and the rich active sites, impressively, Ru‐NiMoO(P)_4_ exhibits superior OER (194 mV @ 50 mA cm^−2^) and HER (24 mV @ 10 mA cm^−2^) activity in alkaline media, far exceeding that of commercial Pt/C and RuO_2_ catalysts. Meanwhile, as bifunctional catalyst, to drive the overall water splitting at the current density of 10 mA cm^−2^, Ru‐NiMoO(P)_4_ requires only 1.45 V and maintaining stable output for 100 h. Furthermore, Ru‐NiMoO(P)_4_ also possesses excellent capability for seawater electrolysis hydrogen production. Moreover, the successful demonstration of wind and solar hydrogen production systems provide the feasibility of the ultra‐low Ru loading catalyst for large‐scale hydrogen production in the future.

## Graphical abstract

A highly active bifunctional catalyst Ru‐NiMoO(P)_4_ is designed and constructed. Due to the synergy of Ru clusters and NiMoO(P)_4_ by the modulated electronic structure and the rich active sites, Ru‐NiMoO(P)_4_ possesses excellent OER, HER and overall water splitting activity in both alkaline media and alkaline seawater, demonstrating excellent application prospect for the wind/solar‐to‐hydrogen generation systems.

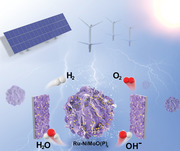

## 1 Introduction

Water electrolysis hydrogen production has the advantages of cleanliness and efficiency, and is of great importance for the future energy economy.^[^ ^1^ ^]^ The development of electrolytic water catalysts adapted to large‐scale industrial applications is essential but challenging.^[^ ^2^ ^]^ Currently, the commercially available catalysts are IrO_2_ for oxygen evolution reaction (OER) and Pt/C for hydrogen evolution reaction (HER), but they are limited by high costs and low stability.^[^ ^3^ ^]^ In addition, most catalysts can only exhibit HER or OER performance in the same medium, which undoubtedly increases the difficulty of industrial hydrogen production.^[^ ^4^ ^]^ Therefore, it is imminent to develop highly cost‐effective bifunctional catalysts.

Ruthenium (Ru), as the cheap platinum group metal with Pt‐like metal hydrogen bond strength and abundant d‐orbital electrons, has good catalytic activity for HER.^[^ ^5^ ^]^ Besides, by appropriate modulation of the electronic structure, Ru can also exhibit outstanding adsorption capabilities of oxygen‐containing intermediates for high‐efficient OER.^[^ ^6^ ^]^ Therefore, it possesses good prospects for application in electrocatalytic monolithic water electrolysis.^[^ ^7^ ^]^ Nevertheless, in practical electrochemical reactions, the catalytic performance gradually decreases as the reaction proceeds due to metal agglomeration and surface remodeling, thus a major concern remains for the durability. Furthermore, oxidation phenomena (from tetravalence to octavalent) and subsequent precipitation of dissolved Ru at high potentials can also lead to deactivation of Ru‐based catalysts.^[^ ^8^ ^]^ Therefore, the design of a suitable substrate material can efficaciously stabilize metallic Ru, which is essential to optimize dispersion issues, reduce particle size, and improve effective active site density and cycling durability.^[^ ^9^ ^]^

In recent years, transition‐metal phosphides (TMPs),^[^ ^10^ ^]^ carbides (TMCs),^[^ ^11^ ^]^ nitrides (TMNs),^[^ ^12^ ^]^ and oxides (TMOs)^[^ ^13^ ^]^ have received widespread attention as substrate materials with high intrinsic activity, abundant raw materials, and strong binding with host materials. Moreover, due to the synergistic effect of metal cations, binary metal compounds tend to show more enhanced electrochemical properties than mono‐metal ones.^[^ ^14^ ^]^ Among them, nickel molybdate (NiMoO_4_) has become a potential substrate material because of easy adjustment of the microstructure and electronic structure.^[^ ^15^ ^]^ However, due to low intrinsic activity, the OER and HER activity of NiMoO_4_ is not ideal.^[^ ^16^ ^]^ Considering that P‐doping can increase the hydrogen binding energy of NiMoO_4_ and promote proton adsorption, the Ru in situ grown on NiMoO(P)_4_ substrate with a larger specific surface area and strong host‐guest interaction would improve the activity and stability of the catalyst.

Based on the above consideration, we design and establish a bifunctional Ru‐NiMoO(P)_4_ catalyst over nickel foam by P‐doping and Ru incorporation. Such a catalyst possesses a nanosheet array structure with large specific surface area and full exposure of active sites. Besides, Ru can adjust the electronic structure of NiMoO(P)_4_, which effectively improves the intrinsic activity. Inspired by the superior OER and HER performance of the catalyst, we further assemble a water electrolysis device. As expected, Ru‐NiMoO(P)_4_ drives water splitting reactions with excellent stability in alkaline and seawater environments. Finally, we combine the water splitting device with photovoltaic power generation and wind power generation. These green energy systems efficiently drive water splitting to produce hydrogen, reduce the external energy consumption for electrolysis of water, and make a significant contribution to the hydrogen economy and the achievement of global carbon neutrality in the near future.^[^ ^17^ ^]^

## 2 Results and Discussion

### 2.1 Synthesis of Catalysts

**Figure 1:**
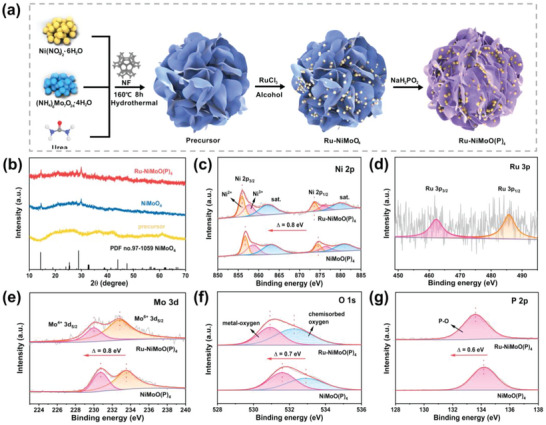
a) Schematic for synthesis of Ru‐NiMoO(P)_4_. b) XRD patterns. XPS spectra of c) Ni 2p, d) Ru 3p, e) Mo 3d, f) O 1s, and g) P 2p.

As shown in **Figure** **1**a, using nickel nitrate and ammonium molybdate tetrahydrate as metal sources, urea as auxiliary agents, nickel foam as substrate, the precursor was synthesized by a hydrothermal method. Subsequently, the precursor was soaked in an ethanol solution containing RuCl_3_·xH_2_O, then followed by P‐doping under low temperature and N_2_ atmosphere. Finally, Ru‐NiMoO(P)_4_/NF was obtained. Furthermore, to explore the effect of Ru etching and P‐doping treatments, Ru‐NiMoO_4_/NF, NiMoO(P)_4_/NF and NiMoO_4_/NF were synthesized in the absence of RuCl_3_·xH_2_O and phosphorus, respectively. The X‐ray diffraction (XRD) pattern of Ru‐NiMoO(P)_4_ is shown in Figure 1b, in which the peaks at 14.3°, 28.9°, and 44.0° are indexed to NiMoO_4_ phase (PDF no.97‐1059), indicating no formation of metal phosphides. However, no characteristic diffraction peak relevant to metallic Ru can be observed for Ru‐NiMoO(P)_4_ except for some dispersion peaks, indicating that the content of Ru in Ru‐NiMoO(P)_4_ is very low, or that Ru is not converted to new phases but exists as clusters or single atoms.

The X‐ray photoelectron spectroscopy (XPS) illustrates that the Ru‐NiMoO(P)_4_ surface consists of Ru, Ni, Mo, P, and O elements (Figure S2, Supporting Information). The peaks located at 855.9 and 873.6 eV are Ni^2+^ 2p_3/2_ and Ni^2+^ 2p_1/2_, respectively, while the peaks at 857.6 and 875.8 eV correspond to Ni^3+^ 2p_3/2_ and Ni^2+^ 2p_1/2_. The two peaks at 862.2 and 879.9 eV are satellite peaks of Ni 2p (Figure 1c).^[^ ^18^ ^]^ The Ru 3p spectrum (Figure 1d) shows two inconspicuous peaks at 462.3 and 485.8 eV, corresponding to Ru 3p_3/2_ and 3p_1/2_ of zero‐valent Ru. The Mo 3d peaks (Figure 1e) at 229.9 and 232.7 eV are attributed to Mo^4+^ 3d_5/2_ and Mo^6+^ 3d_5/2_ of Ru‐NiMoO(P)_4_, respectively.^[^ ^19^ ^]^ The two peaks in the O 1s spectrum (Figure 1f) indicate the presence of metal–oxygen bonds (530.9 eV) and chemically adsorbed oxygen (532.3 eV) on the surface. For the P 2p spectrum (Figure 1g; Figure S3, Supporting Information), the peak at 134.2 eV is a P─O bond and no peaks of metal phosphides appear, further indicating the replacement of oxygen by P in NiMoO_4_ during the process of phosphorylation and Ru incorporation, and no generation of metal phosphides.^[^ ^20^ ^]^ The change of XPS spectra after P‐doping (Figure S4, Supporting Information) proves that the lower electronegativity of P than that of O leads to the successful P‐doping into NiMoO_4_. This demonstrates the beneficial electron transfer and charge density redistribution caused by ion exchange, resulting in a decreased intermediate energy of the reaction.^[^ ^21^ ^]^ Noteworthily, the Ni 2p and Mo 3d peaks of Ru‐NiMoO(P)_4_ show significant negative shifts relative to NiMoO(P)_4_, indicating the overall shift is caused by the electron transfer from Ru to Ni/Mo sites in Ru‐NiMoO(P)_4_, which increases the electrical conductivity and intrinsic activity of the catalyst.^[^ ^22^ ^]^ Meanwhile, after Ru etching and P‐doping, only the Mo 3d pattern has a negative shift, which results from the synergistic effect of Ru and P anions.

**Figure 2:**
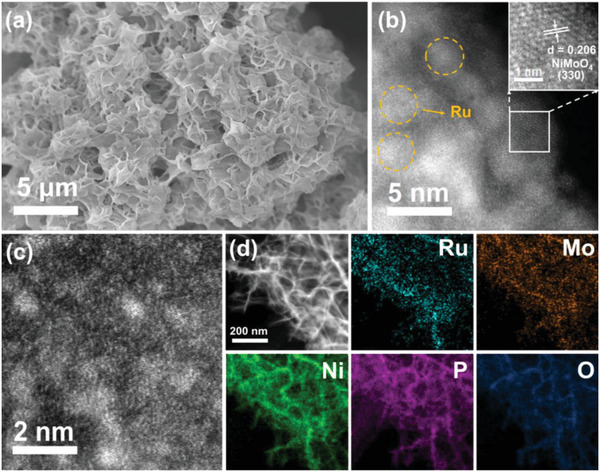
a) SEM image of Ru‐NiMoO(P)_4_. b‐c) HAADF‐STEM images of Ru‐NiMoO(P)_4_. d) HAADF‐STEM image and EDX elemental mapping of Ru‐NiMoO(P)_4_.

From the scanning electron microscopy (SEM) images, it can be observed that Ru‐NiMoO(P)_4_ nanosheet arrays uniformly grow on NF substrate (**Figure** **2**a). The SEM images of the precursor and catalyst (Figures S5 and S6, Supporting Information) show that after Ru impregnation and P‐doping, the nanosheets of the samples change slightly, indicating no significant influence on the morphology of the catalyst. The N_2_ adsorption and desorption test results (Figure S7, Supporting Information) demonstrate that the 3D sheet structure consisting of a large number of very thin nanosheets provides a larger surface area for the catalyst, which facilitates the increase of catalytic sites and electron transfer. To further explore the nano structure of Ru‐NiMoO(P)_4_, we performed an in‐depth analysis using spherical aberration‐corrected scanning transmission electron microscopy (ac‐STEM). The clear lattice fringe with the spacing of 0.206 nm (Figure 2b) corresponds to the (330) crystal plane of NiMoO_4_, which is consistent with the XRD analysis result, while it is absent for the lattice fringe of Ru. As shown in Figure 2c, the bright Ru can be observed on the relatively dark substrate material, further demonstrating the presence of Ru clusters. Besides, the corresponding positions of Ni and O elements can be seen in the energy dispersive X‐ray (EDX) spectrum (Figure S8, Supporting Information). This is consistent with the inductively coupled plasma optical emission spectroscopy (ICP‐OES) test result of 2.57 wt.% Ru (Figure S1, Supporting Information). Furthermore, the high‐angle annular dark‐field (HAADF) STEM image and EDX mapping (Figure 2d; Figure S9, Supporting Information) verify the distribution of Ru, Ni, Mo, O, and P elements on the nanosheet. Significantly, the P is dispersed with Ni, Mo, and O, which also proves that P was doped into nickel molybdate, consistent with the previous XPS analysis results. Above characterization results demonstrate the successful synthesis of Ru‐NiMoO(P)_4_.

### 2.2 Electrocatalytic Performance Toward OER and HER

**Figure 3:**
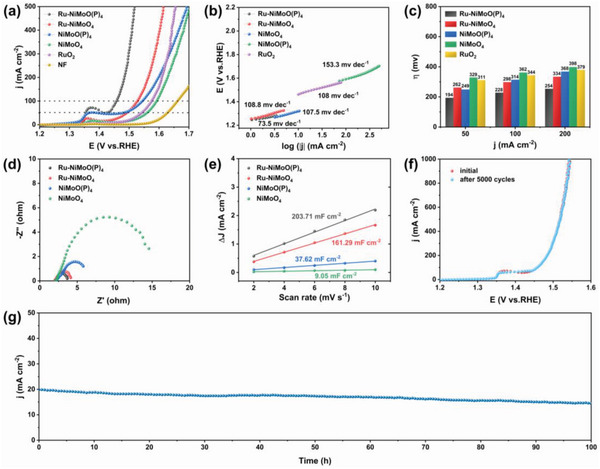
a) OER polarization curves, b) Tafel slopes, and c) corresponding overpotentials of various catalysts in alkaline freshwater media. d) Nyquist plots. e) The plots of the current density versus the scan rate of various catalysts. f) LSV curves obtained initial and after 5000 CV cycles. g) Time‐reliant current density curve.

The OER performance of Ru‐NiMoO(P)_4_, Ru‐NiMoO_4_, NiMoO(P)_4_, NiMoO_4_, and RuO_2_ on NF and bare NF was investigated in 1 m KOH freshwater. As depicted in **Figure** **3**a, the activity trend of Ru‐NiMoO(P)_4_ > Ru‐NiMoO_4_ > NiMoO(P)_4_ > NiMoO_4_ > RuO_2_ on NF > bare NF indicates the improvement of OER activity by Ru‐cluster incorporation and subsequent P‐doping. Among them, Ru‐NiMoO(P)_4_ exhibits superior catalytic activity to the commercial RuO_2_ catalyst. Moreover, it requires only 194 and 228 mV to achieve current densities of 50 and 100 mA cm^−2^, respectively (Figure 3c), which far exceed Ru‐NiMoO_4_ (262 and 298 mV), NiMoO(P)_4_ (249 and 314 mV), NiMoO_4_ (329 and 362 mV), and RuO_2_ (311 and 344 mV). At larger current densities, Ru‐NiMoO(P)_4_ still performs better than control samples. Notably, its OER activity is significantly better than that of NiMoO(P)_4_, suggesting that the presence of Ru clusters does facilitate the OER activity. The excellent OER performance is also superior to the recently reported noble metal catalysts (Table S3, Supporting Information).

In addition, the Tafel slopes were calculated from the LSV curves. As depicted in Figure 3b, Ru‐NiMoO(P)_4_ has the smallest Tafel slope (73.5 mV dec^−1^) compared with control samples, indicating the optimal electrocatalytic kinetic mechanism. Besides, the double‐layer capacitance (C_dl_) was calculated by the cyclic voltammetry (CV) curves at continuous scanning speed in the non‐Faraday region (Figure S10, Supporting Information). The C_dl_ value of Ru‐NiMoO(P)_4_ (203.71 mF cm^−2^) is much higher than those of Ru‐NiMoO_4_ (161.29 mF cm^−2^), NiMoO(P)_4_ (37.62 mF cm^−2^) and NiMoO_4_ (9.05 mF cm^−2^), as shown in Figure 3e. Subsequently, to further investigate the reason for the improved activity of Ru‐NiMoO(P)_4_, the C_dl_ is used to reflect the electrochemically active surface area (ECSA) (Table S1, Supporting Information), and the ECSA normalized polarization curve reveals that Ru‐NiMoO(P)_4_ has the highest intrinsic activity (Figure S22, Supporting Information). Therefore, the presence of Ru clusters in the Ru‐NiMoO(P)_4_ sheet structure effectively increases the active sites, charge transfer rate, and intrinsic activity. Meanwhile, the reaction kinetics of various catalysts was investigated using electrochemical impedance spectroscopy (EIS). As expected, Ru‐NiMoO(P)_4_ reveals a minimum charge transfer resistance (R_ct_), indicating extremely fast charge transfer capability (Figure 3d).

Besides, the OER durability of Ru‐NiMoO(P)_4_ is another important parameter. As shown in Figure 3f, the LSV curve of Ru‐NiMoO(P)_4_ shifts slightly before and after 5000 cycles. In addition, the results of the chronoamperometric tests (Figure 3g) show that the catalyst can maintain stable catalysis for 100 h. These confirm the excellent long‐term stability of Ru‐NiMoO(P)_4_. Besides, the XPS and Raman spectroscopy analysis of Ru‐NiMoO(P)_4_ after OER confirms the surface oxidation. As shown in Figure S11a,b (Supporting Information), the positive peak shift of Ni 2p and Mo 3d indicates that Ni and Mo were oxidized during the OER process. The spectrum of Mo 3d shows two obvious peaks at 233.7 and 235.8 eV corresponding to Mo^4+^ 3d_3/2_ and Mo^6+^ 3d_3/2_, which demonstrates that Mo mainly exists in the form of molybdenum oxides.^[^ ^23^ ^]^ Meanwhile, the Ru 3p spectrum (Figure S11c, Supporting Information) shows that the peak of Ru is slightly negatively shifted, indicating the possible formation of an oxide layer on the Ru cluster. In addition, Raman spectroscopy further demonstrates the formation of metal oxides. Two new characteristic peaks (Figure S12, Supporting Information) appear at 474.1 and 544.3 cm^−1^, corresponding to the Ni─O vibration pattern, indicating the formation of NiOOH.^[^ ^24^ ^]^ These oxides and hydroxides continue to drive the reaction as additional active sites, reducing the adsorption energy of the intermediates and improving the OER performance of Ru‐NiMoO(P)_4_. Based on the above analysis, excellent electrocatalytic performance of Ru‐NiMoO(P)_4_ can be attributed to Ru‐cluster coupling, 3D sheet structures, P‐doping, high charge transfer speed, abundant active sites and mechanical durability.

**Figure 4:**
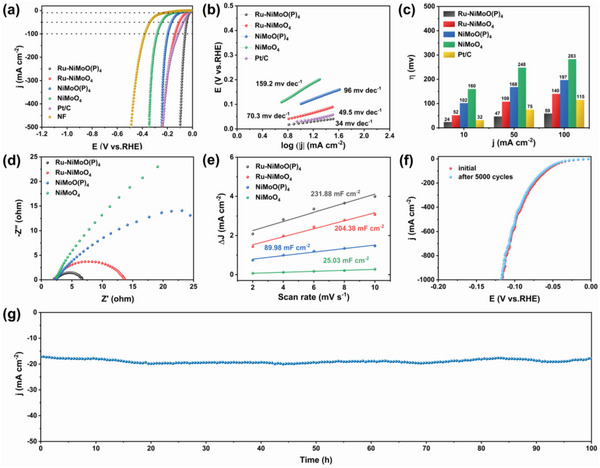
a) HER polarization curves, b) Tafel slopes, and c) corresponding overpotentials of various catalysts in alkaline freshwater. d) Nyquist plots. e) The plots of the current density versus the scan rate of various catalysts. f) LSV curves obtained initial and after 5000 CV cycles. g) Time‐reliant current density curve.

The HER activity of these catalysts was also investigated in alkaline freshwater. As shown in **Figure** **4**a, the HER activity of Ru‐NiMoO(P)_4_ is significantly higher than that of NiMoO(P)_4_, indicating that the introduction of Ru is indeed beneficial to promote the HER activity. Impressively, as presented in Figure 4c, Ru‐NiMoO(P)_4_ requires only the overpotential of 24 mV to achieve the current density of 10 mA cm^−2^, much lower than that of commercial Pt/C catalysts (32 mV). Such excellent activity is also quite competitive among the recently reported nano catalysts (Table S4, Supporting Information). Furthermore, Ru‐NiMoO(P)_4_ exhibits a low Tafel slope (34 mV dec^−1^), indicating rapid HER kinetics (Figure 4b).^[^ ^25^ ^]^ As shown in Figure 4d, Ru‐NiMoO(P)_4_ exhibits the smallest R_ct_ of 4.2 Ω, demonstrating the fastest charge transfer rate. In addition, Ru‐NiMoO(P)_4_ has a greater C_dl_ value than other catalysts (Figure 4e; Figure S13, Supporting Information), and the ECSA normalized polarization curves of HER (Figure S22, Supporting Information) also indicate that Ru‐NiMoO(P)_4_ has the highest intrinsic catalytic activity. Figure 4f shows that the LSV curve of Ru‐NiMoO(P)_4_ before and after 5000 cycles are essentially the same. Meanwhile, the results of the chronoamperometry tests (Figure 4g) show that the catalyst is stable for 100 h, without obvious performance degradation. As shown in Figure S14 (Supporting Information), the spectrum of Ni 2p has no obvious change, proving no change for the surface Ni composition. Meanwhile, the Ru 3p peak apparently declines, indicating that Ru precipitation occurs during the HER process. The positive peak shift of Mo 3d and the negative shift of P 2p can be attributed to the reduction reaction during the HER process, which indicates that Mo is an active species for the electrochemical HER.^[^ ^26^ ^]^ In addition, no significant changes can be observed in the Raman spectra (Figure S15, Supporting Information) before and after HER, further demonstrating the robust HER activity and stability of Ru‐NiMoO(P)_4_ in alkaline electrolytes.

### 2.3 Seawater Electrolysis and Hydrogen Generation Systems

**Figure 5:**
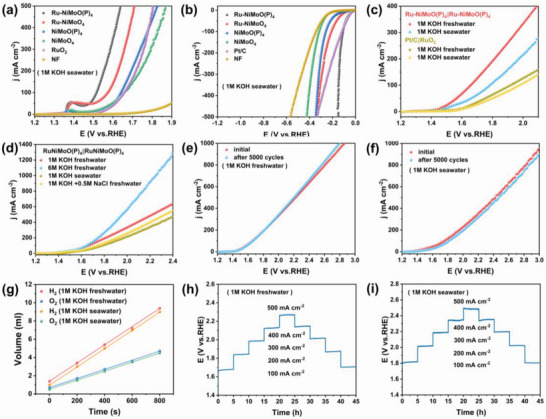
a) OER and b) HER polarization curves of various catalysts in alkaline seawater. c) Polarization curves of Ru‐NiMoO(P)_4_ || Ru‐NiMoO(P)_4_ and Pt/C on NF || RuO_2_ on NF in alkaline freshwater and in alkaline seawater. d) Polarization curves of Ru‐NiMoO(P)_4_ || Ru‐NiMoO(P)_4_ pair in various alkaline media. LSV curves obtained initial and after 5000 CV cycles e) in alkaline freshwater and f) in alkaline seawater. g) The volume of H_2_ and O_2_ was experimentally measured versus time. Multicurrent chronopotentiometry response for Ru‐NiMoO(P)_4_ in h) alkaline freshwater and i) alkaline seawater.

To alleviate the dependence on freshwater resources, the catalytic performance of the samples in alkaline seawater media was explored. As shown in **Figure** **5**a and Figure S16 (Supporting Information), Ru‐NiMoO(P)_4_ presents excellent OER activity with only 250 and 286 mV to achieve the current density of 50 and 100 mA cm^−2^, respectively, superior to the RuO_2_ on NF in seawater (382 and 427 mV). Meanwhile, as revealed in Figure 5b and Figure S18 (Supporting Information), it also possesses outstanding HER activity in seawater, providing overpotentials as low as 37 and 117 mV at current densities of 10 and 100 mA cm^−2^, respectively, comparable to that of commercial Pt/C catalysts on NF (35 and 119 mV). In addition, Ru‐NiMoO(P)_4_ owns a lower Tafel slope (Figures S17 and S19, Supporting Information), indicating good reaction kinetics even in seawater. Besides, the results of the chronoamperometric test (Figures S20 and S21, Supporting Information) show that, in seawater, its HER and OER processes are very stable, without obvious activity degradation for 100 h. Notably, the OER/HER activity of Ru‐NiMoO(P)_4_ is quite competitive as compared with those recently reported noble metal catalysts (Table S6, Supporting Information), even in seawater media.

Subsequently, the H‐type electrolytic cell was assembled to evaluate the overall water splitting (OWS) property of Ru‐NiMoO(P)_4_. The Ru‐NiMoO(P)_4_ || Ru‐NiMoO(P)_4_ pair requires merely 1.45 V to obtain the current density of 10 mA cm^−2^, exhibiting outstanding OWS performance in alkaline freshwater, which also exceeds the Pt/C || RuO_2_ pair (1.58 V) and the recently reported catalysts (Figure 5c; Figure S26 and Table S5, Supporting Information). Besides, in seawater, the Ru‐NiMoO(P)_4_ || Ru‐NiMoO(P)_4_ pair also reveals excellent OWS activity (1.48 V @ 10 mA cm^−2^), as presented in Figure S26 (Supporting Information). Then to reach 100 mA cm^−2^, the Ru‐NiMoO(P)_4_ || Ru‐NiMoO(P)_4_ pair only needs 1.66 and 1.78 V in alkaline freshwater and alkaline seawater, respectively. Meanwhile, Figure 5d reflects that the catalyst maintains excellent OWS performance in a variety of electrolytes. Overall, the presence of insoluble precipitation, bacteria, and microbes in seawater can inactivate the active site, leading the relatively poor catalysis performance in alkaline seawater compared to the OWS performance in alkaline media.^[^ ^27^ ^]^ Moreover, Figure S27 (Supporting Information) depicts that the Tafel slope of Ru‐NiMoO(P)_4_ || Ru‐NiMoO(P)_4_ in both alkaline freshwater and alkaline seawater is smaller than the commercial Pt/C || RuO_2_ pair, proving its high hydrolysis kinetic activity.

In addition, the Faraday efficiency of water splitting on Ru‐NiMoO(P)_4_/NF electrodes was calculated by collecting hydrogen and oxygen produced at both sides of the alkaline electrolytic cell by the drainage method (Figures S30 and S31, Supporting Information). Figure 5g reveals that the volume ratio of H_2_ to O_2_ is about 2:1, indicating that the Faraday efficiency is ≈ 100% in alkaline freshwater and alkaline seawater. Besides, the results of CV (Figure 5e,f) and chronoamperometry test (Figures S28 and S29, Supporting Information) also suggest that the Ru‐NiMoO(P)_4_ || Ru‐NiMoO(P)_4_ pair only has a weak performance degradation in seawater. Meanwhile, the multi‐step timing potential test (Figure 5h,i) shows that the catalyst possesses a stable gradient trend at different current densities, maintaining almost the same potential after reaching 500 mA cm^−2^ at the same current density (500–100 mA cm^−2^). Undoubtedly, it proves that the catalyst has excellent charge transport capability and durability. All the above results demonstrate that the Ru‐NiMoO(P)_4_ can be used for seawater electrolysis, as good alternative for electrocatalytic hydrogen production.

Thus, the excellent electrocatalytic performance of Ru‐NiMoO(P)_4_ can be attributed to the following aspects: First of all, the introduction of Ru optimizes the electronic structure of the NiMoO(P)_4_ array, increases the density of the active sites and the intrinsic activity, and accelerates the charge transfer of Ru‐NiMoO(P)_4_. Second, the electron synergistic effect of Ru and P anions further promotes charge transfer, conducive to accelerating electrochemical kinetics. In addition, the 3D sheet structure composed of a large number of ultra‐thin nanosheets provides more active sites and a larger specific surface area, promoting the mass transfer. Also, the unique structure loaded on nickel foam with good electrical conductivity can efficiently transfer mass and electrons during the water electrolysis process.

**Figure 6:**
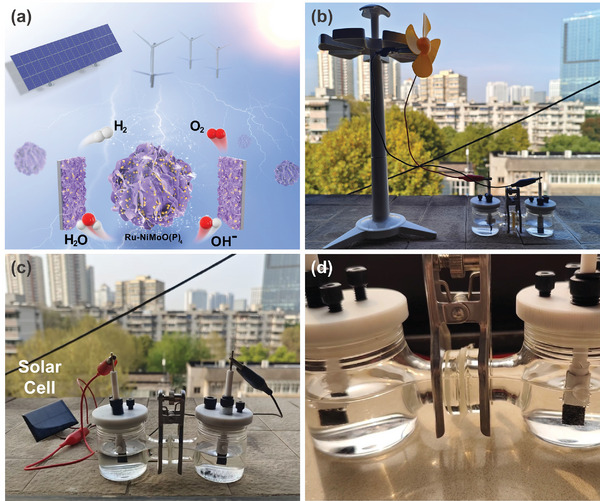
a) Schematic illustration of the integrated wind‐to‐hydrogen and solar‐to‐hydrogen system. b) Digital photograph of the integrated wind‐to‐hydrogen system and c) solar‐to‐hydrogen system. d) Photograph of bubbles attached to NF.

For practical applications, hydrogen production by water electrolysis can use the abundant renewable energy in nature to further reduce costs, protect the environment, and realize the concept of “green hydrogen energy”. Then the water electrolysis hydrogen production device can be assembled with a wind power device, using the wind to drive the wind turbine to rotate and generate electricity to drive the water electrolysis hydrogen production (**Figure** **6**b). As shown in Video S1 (Supporting Information), the bubbles can be observed on both sides of the electrolytic cell, proving successfully hydrogen and oxygen production using the catalyst electrode. Meanwhile, since solar energy is renewable, the water electrolysis can be combined with the photovoltaic industry to design an integrated system connecting solar cell and water electrolysis devices to drive electrocatalytic hydrogen production by abundant solar energy (Figure 6c). Figure 6d and Video S2 (Supporting Information) show that the violent gas can be generated on both sides of the electrolytic cell, indicating that the device has a high efficiency in hydrogen production and a promising potential application. To sum up, employing natural renewable wind and solar energy sources to produce hydrogen can avoid the limitations of electrical energy. Undoubtedly, it offers the hope for actual large‐scale applications of Ru‐NiMoO(P)_4_ catalysts in electrochemical hydrogen production.

## 3 Conclusion

In summary, the bifunctional catalyst, Ru‐NiMoO(P)_4_/NF, with a unique nanosheet structure, is synthesized by hydrothermal, Ru incorporation and P‐doping. The Ru cluster and NiMoO(P)_4_ jointly regulate the electronic structure, improve intrinsic activity, and provide additional active sites. Ru‐NiMoO(P)_4_/NF exhibits superior OER (194 mV @ 50 mA cm^−2^) and HER (24 mV @ 10 mA cm^−2^) performance in alkaline media. The OWS performance of the catalyst is also excellent, requiring only 1.45 V to achieve the current density of 10 mA cm^−2^, with almost 100% Faraday efficiency in alkaline media. Besides, Ru‐NiMoO(P)_4_ achieves the current density of 10 mA cm^−2^ at only 1.48 V in seawater. Moreover, we demonstrate the water electrolysis with solar/wind‐powered hydrogen production systems, building a feasible idea for future large‐scale hydrogen production from water electrolysis.

## 4 Experimental Section

### Treatment of Foam Nickel

Nickel foam was sonicated with the diluted hydrochloric acid solution, deionized (DI) water, and ethanol solution for 10 min, respectively.

### Synthesis of Ru‐NiMoO(P)_4_/NF

0.9 mmol Ni(NO_3_)_2_·6H_2_O, 0.135 mmol (NH_4_)_6_Mo_7_O_24_·4H_2_O and 2 mmol urea were dissolved in 30 mL DI water and stirred for 15 min at room temperature. Afterward, the mixed solution with the pretreated NF was transferred to a 50 mL Teflon‐lined autoclave and kept at 160 °C for 8 h. The obtained precursor was washed with DI water and dried at 60°C. Subsequently, the precursor was immersed into an ethanol solution (50 mL) containing RuCl_3_·xH_2_O (100 mg) at room temperature. After reaction for 15 min, the sample was taken out and dried at 60 °C. Then, NaH_2_PO_2_·H_2_O (1.0 g) and the sample were put at the upstream side and center of the tube furnace, respectively. The sample was then annealed at 350 °C for 2 h in an inert atmosphere. Finally, Ru‐NiMoO(P)_4_/NF was obtained after cooling down to room temperature.

## Conflict of Interest

The authors declare no conflict of interest.

## Supporting information

Supporting Information

Supplemental Video 1

Supplemental Video 2

## Data Availability

Research data are not shared.
