# Supplementary material for: Ru Cluster Incorporated NiMoO(P)4 Nanosheet Arrays as High‐Efficient Bifunctional Catalyst for Wind/Solar‐To‐Hydrogen Generation Systems
Source: Adv Sci (Weinh). 2023 Oct 25;10(35):2304179. doi: 10.1002/advs.202304179 (PMC10724388; doi:10.1002/advs.202304179)
Supplement: Supplementary file 1 — Supporting Information [file ADVS-10-2304179-s001.pdf]

ADVANCED SCIENCE
Open Access

Supporting Information

for *Adv. Sci.*, DOI 10.1002/advs.202304179

Ru Cluster Incorporated $NiMoO(P)_4$ Nanosheet Arrays as High-Efficient Bifunctional Catalyst for Wind/Solar-To-Hydrogen Generation Systems

Shengye Wu, Ding Chen, Shang Li, Yuting Zeng, Tao Wang, Jian Zhang, Jun Yu, Shichun Mu* and Haolin Tang**

Supporting Information

Ru cluster incorporated $NiMoO(P)_4$ nanosheet arrays as high-efficient bifunctional catalyst for wind/solar-to-hydrogen generation systems

Shengye Wu[1,2], Ding Chen[1], Shang Li[1], Yuting Zeng[1], Tao Wang[1], Jian Zhang[1], Jun Yu[*1,2], Shichun Mu[*1,2], Haolin Tang[*1,2]

[1]*State Key Laboratory of Advanced Technology for Materials Synthesis and Processing, Wuhan University of Technology, Wuhan 430070, China.*

[2]*Key Laboratory of Fuel Cell Technology of Hubei Province, Wuhan University of Technology, 430070, China.*

** Corresponding author E-mail: yujun@whut.edu.cn; msc@whut.edu.cn; thln@whut.edu.cn*

1. Experimental Details

1.1 Materials and Reagents

Nickel nitrate hexahydrate ($Ni(NO_3)_2 \cdot 6H_2O$), ammonium molybdate tetrahydrate ($(NH_4)_6Mo_7O_{24} \cdot 4H_2O$), urea and sodium hypophosphite ($NaH_2PO_2 \cdot H_2O$) were obtained from Sinopharm Group Chemical Reagent. Potassium hydroxide (KOH), absolute ethanol, and hydrochloric acid were purchased from Beijing Chemical Works. RuO_2, Pt/C (20 wt%) powder, and Nafion solution (5 wt%) were purchased from Sigma-Aldrich. Ruthenium chloride hydrate ($RuCl_3 \cdot xH_2O$) was purchased from Aladdin. All the reagents are analytical grade and used without further treatment. Deionized (DI) water was employed as solvent. Natural seawater was obtained from the Yellow Sea of China and filtered for further use.

1.2 Material Characterization

X-ray diffraction (XRD) patterns were collected on a Bruker D8-Advance X-ray diffractometer equipped with a Cu $K\alpha$ radiation source. The morphology and structure were characterized by scanning electron microscopy (SEM, FEI nanoSEM450) and double spherical aberration-corrected scanning transmission electron microscope (AC-STEM, Titan Cubed Themis G2 300). The chemical state of samples shown in XPS images was performed on an ESCALAB-250Xi spectrometer equipped with monochromatic Al Ka radiation. The Raman spectrum is tested on the Renishaw Raman Microscope (LabRAM Odyssey) to determine hydroxyl oxides. ICP-OES analysis was performed on Optima Prodigy 7.

1.3 Electrochemical Measurements

All electrochemical measurements were performed in a conventional three-electrode system at room temperature using a CHI 660E electrochemical analyzer (CHI Instruments, Shanghai, China). A graphite rod and Hg/HgO were used as the counter electrode and the reference electrode, respectively. The as-prepared electrocatalysts with a geometric area of 0.25 cm^2 were directly served as the working electrodes. As for powdery catalysts (RuO_2 and Pt/C), the working electrodes were prepared by dropping electrocatalyst ink onto NF. The alkaline freshwater and seawater media were prepared by adding potassium hydroxide to freshwater and seawater (1M KOH, pH≈14), respectively. The scan rate of the polarization curves of HER and water splitting was 5 mV s^{-1}, but OER was 1 mV s^{-1} in order to reduce the effect of nickel oxidation peak. In HER and OER characterizations, the polarization curves were iR-corrected using the equation: $E_{iR\text{-corrected}} = E - iR$, where E is the original potential, R is the solution resistance, i is the corresponding current, and $E_{iR\text{-corrected}}$ is the iR-corrected potential. Electrochemical impedance spectroscopy (EIS) tests were carried out in a frequency ranging from 0.01 Hz to 100 kHz with AC amplitude of 10 mV at the voltage corresponding to 300 mV overpotential (OER) and 100 mV overpotential (HER). Furthermore, the HER and OER potentials were converted to RHE scale according to the equation: E (vs. RHE) = E (vs. Hg/HgO) + 0.059*pH + 0.098 V. The electrochemical double layer capacitance (C_{dl}) was determined with typical cyclic voltammetry (CV) measurements at various scan rates (2, 4, 6, 8 and 10 mV s^{-1}) in 0.1 ~ 0.2 V (for OER) and (-0.87) ~ (-0.77) V (for HER) versus Hg/HgO. The C_{dl} can be further converted into ECSA using the specific capacitance value for a standard with 1

cm^{-2} of real surface area. The specific capacitance for a flat surface is normally between 0.02-0.06 mF cm^{-2}. But the capacitance value of Ni foam is much larger than that of a flat surface, so we have added the C_{dl} value of the bare NF (2.0 mF cm^{-2}) and used this as a standard to estimate the ECSA. [26] The calculation formula is as follows：

$$X_{ECSA} = \frac{C_{dl}(catalyst)\ mF\ cm^{-2}}{C_{dl}(NF) mF\ cm^{-2} \cdot per\ ECSA\ cm^{-2}}$$

The electrocatalyst Ru-NiMoO(P)$_4$/NF was used as both cathode and anode in a two-electrode configuration for overall water splitting. Notably, the generated H_2 and O_2 gases during overall water splitting were quantitatively collected by the water drainage method.

2. Supplementary Figures and Tables

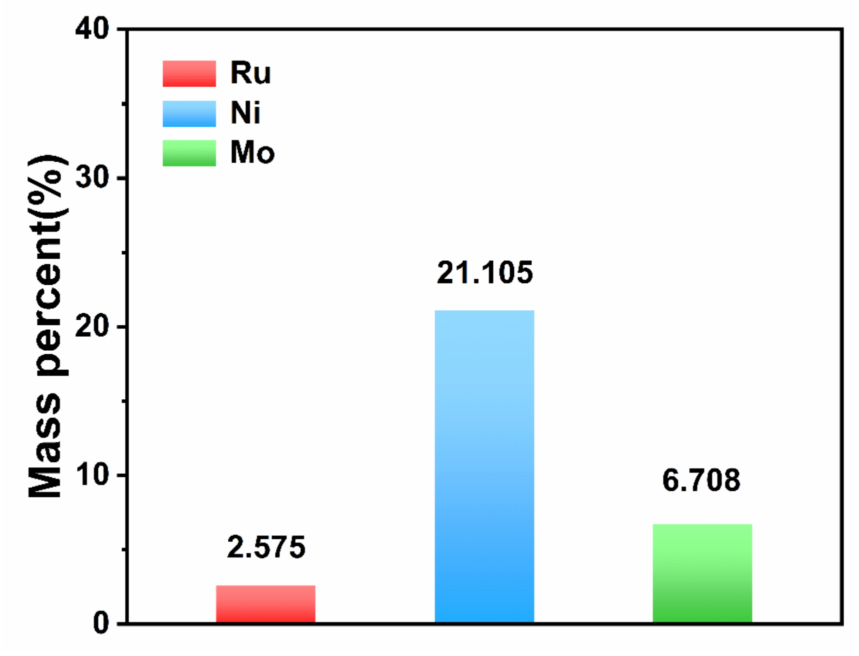

Figure S1. Ni, Mo and Ru atomic percentages in ICP-OES.

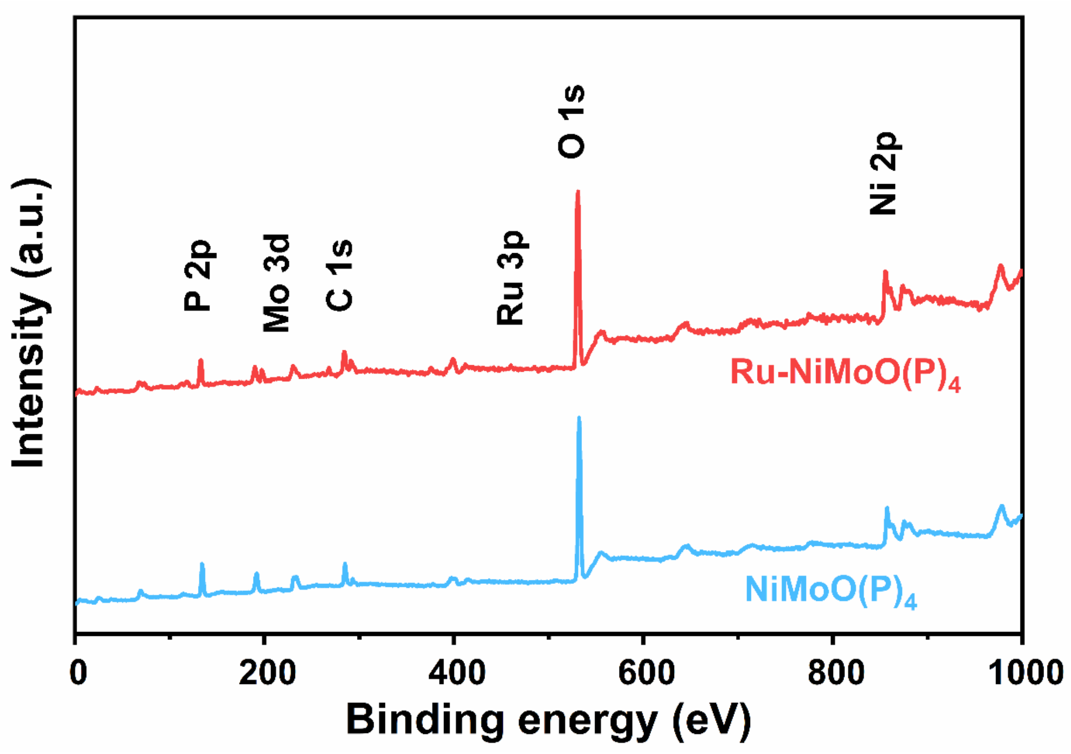

Figure S2. XPS full spectra of Ru-NiMoO(P)$_4$.

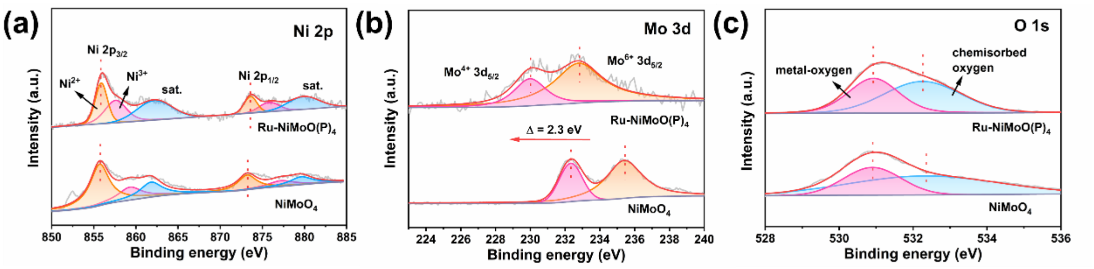

Figure S3. XPS spectra of Ru-$NiMoO(P)_4$ and $NiMoO_4$ in the a) Ni 2p, b) Mo 3d, and c) O 1s region.

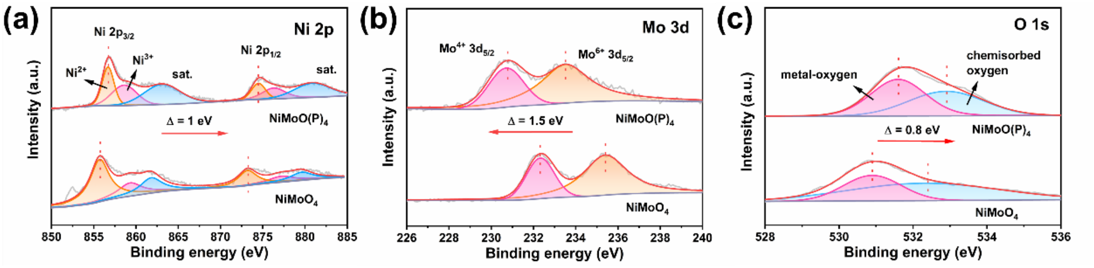

Figure S4. XPS spectra of $NiMoO(P)_4$ and $NiMoO_4$ in the a) Ni 2p, b) Mo 3d, and c) O 1s region.

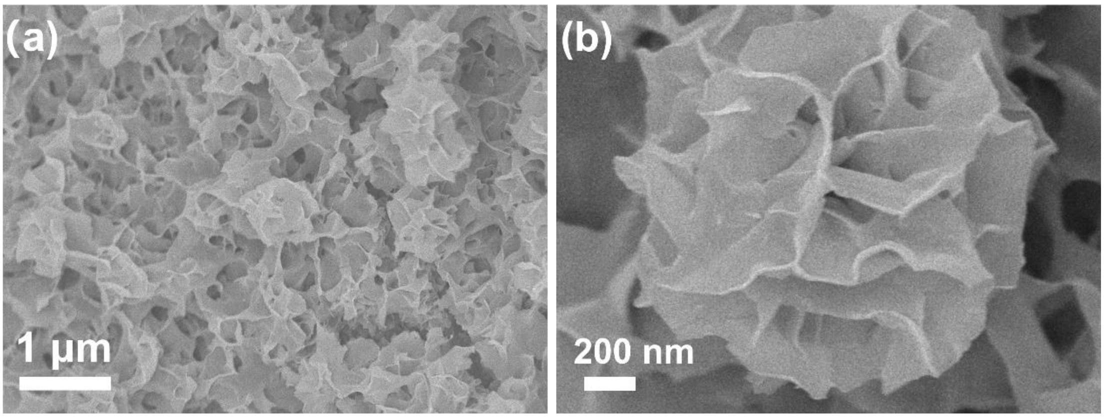

Figure S5. SEM images of precursor a) and b).

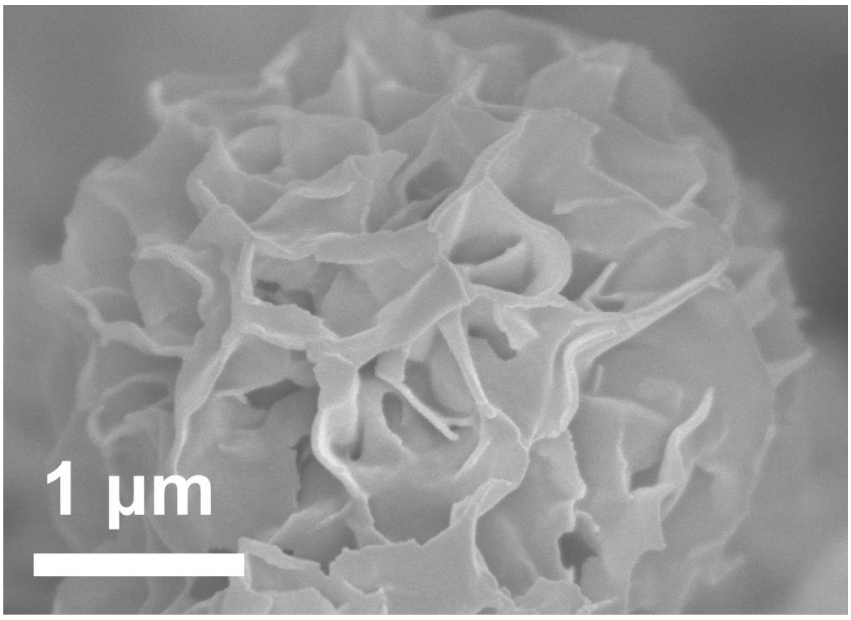

Figure S6. SEM image of Ru-NiMoO(P)$_4$.

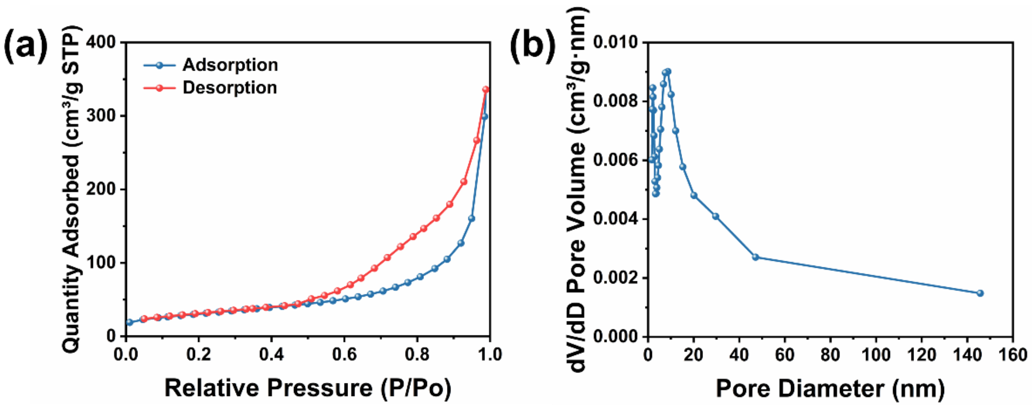

Figure S7. a) N_2 adsorption and desorption isotherms of Ru-NiMoO(P)$_4$. b) Pore size distribution curves of Ru-NiMoO(P)$_4$.

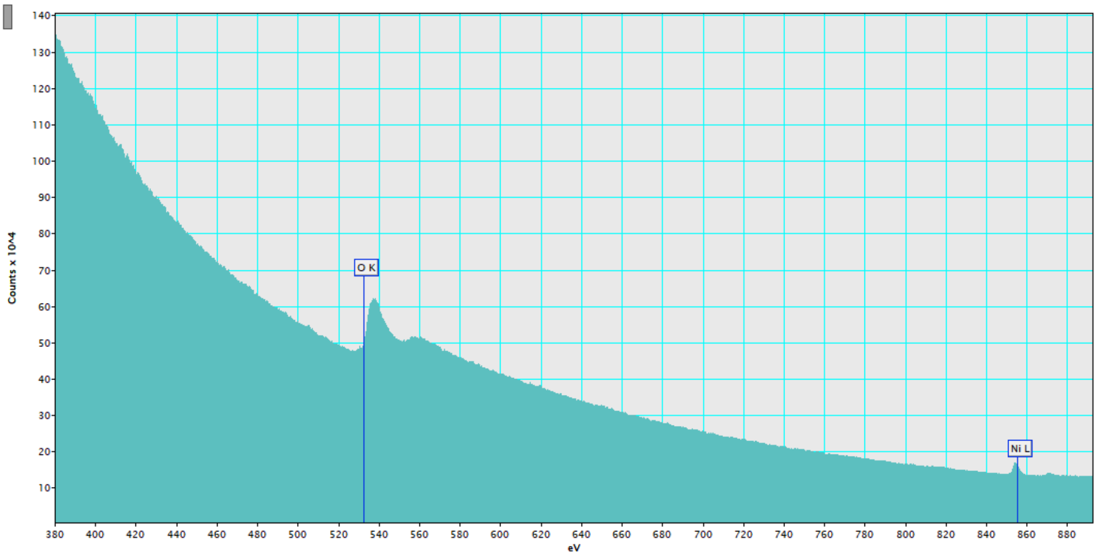

Figure S8. EDX spectrum of Ru-NiMoO(P)$_4$.

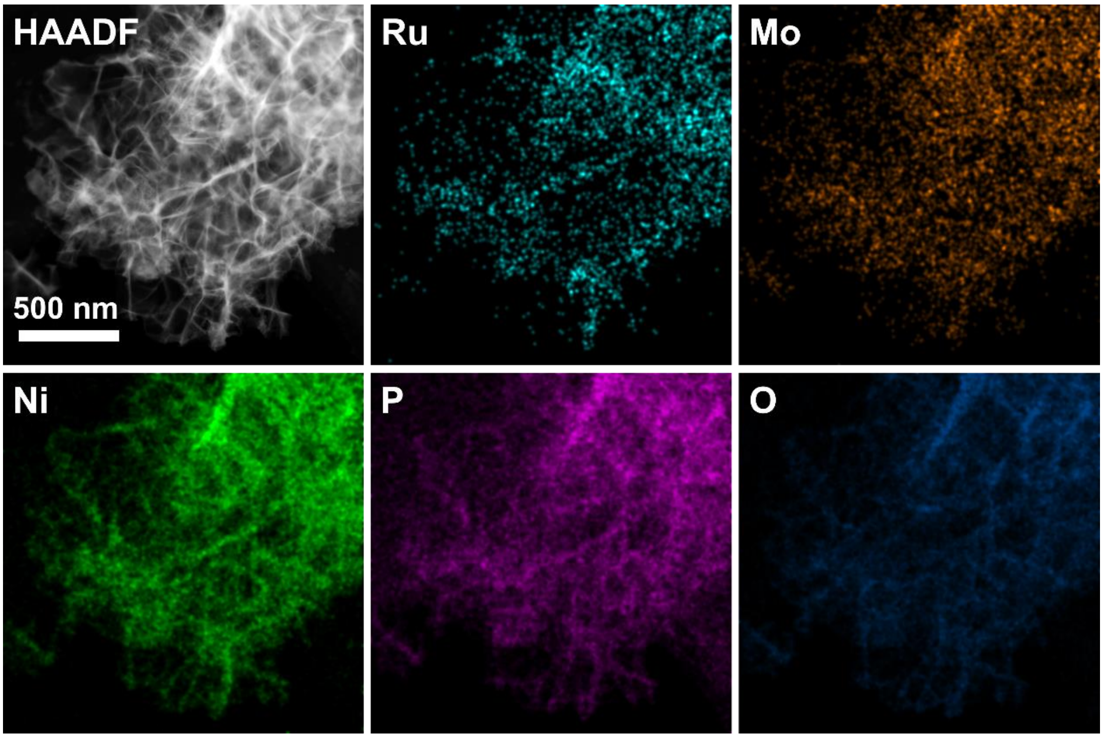

Figure S9. EDX elemental mapping images for Ru, Mo, Ni, P and O in Ru-NiMoO(P)$_4$/NF.

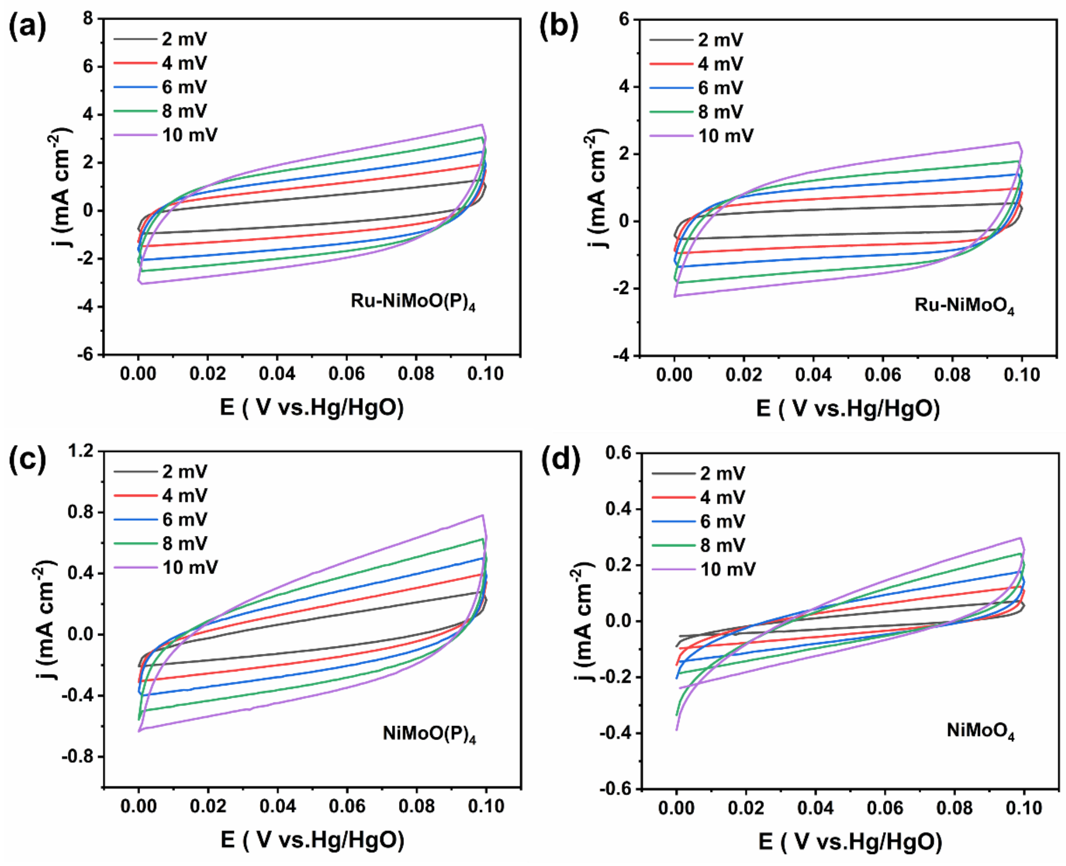

Figure S10. CV curves of a) Ru-$NiMoO(P)_4$/NF, b) Ru-$NiMoO_4$/NF, c) $NiMoO(P)_4$/NF and d) $NiMoO_4$/NF at scan rates ranging from 2 to 10 mV s^{-1} outside OER region.

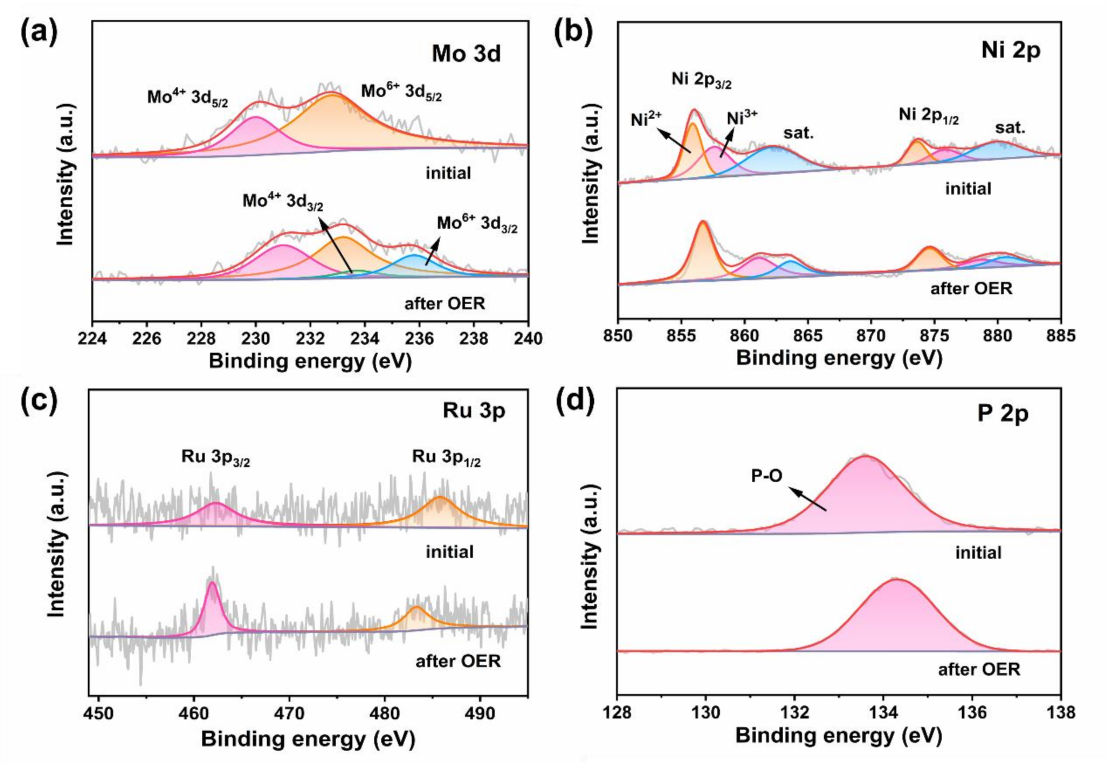

Figure S11. XPS spectra for a) Mo 3d, b) Ni 2p, c) Ru 3p, and d) P 2p of Ru-NiMoO(P)$_4$ before and after OER test.

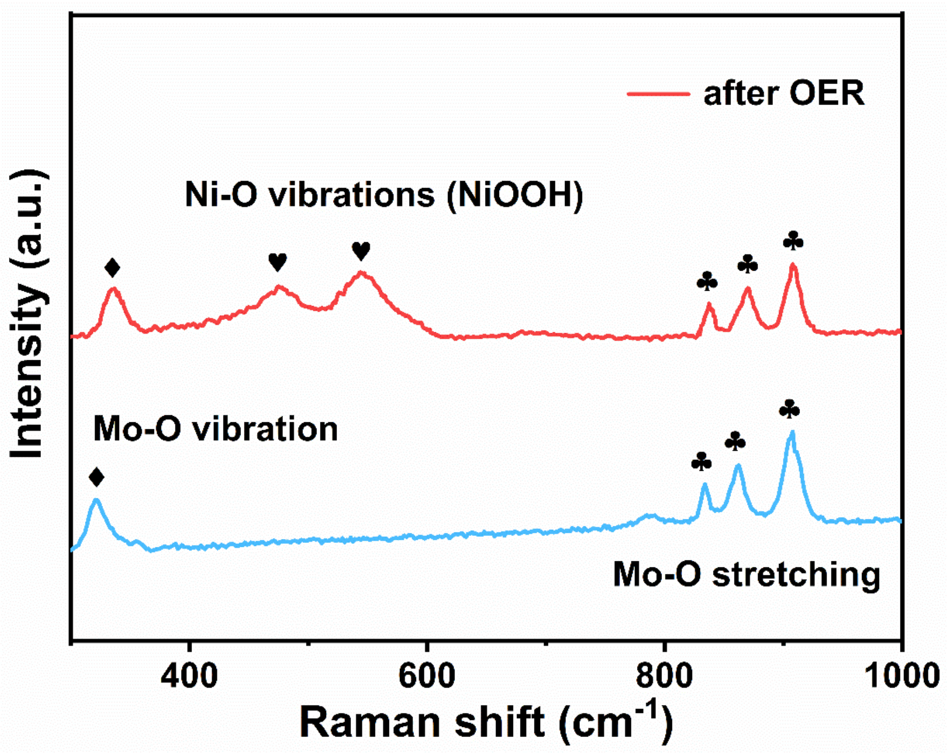

Figure S12. Raman spectra of Ru-NiMoO(P)$_4$ before and after OER test.

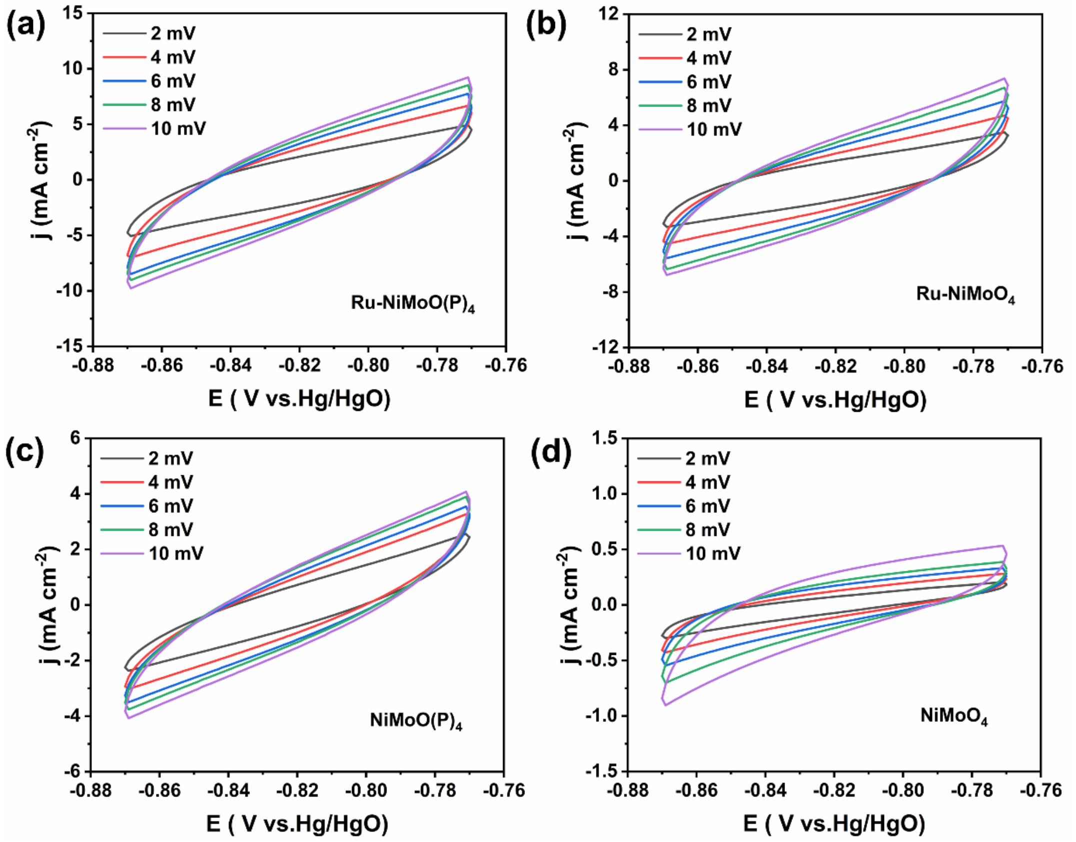

Figure S13. CV curves of a) Ru-NiMoO(P)$_4$/NF, b) Ru-NiMoO(P)$_4$/NF /NF, c) NiMoO(P)$_4$/NF and d) NiMoO$_4$/NF at scan rates ranging from 2 to 10 mV s^{-1} outside HER region.

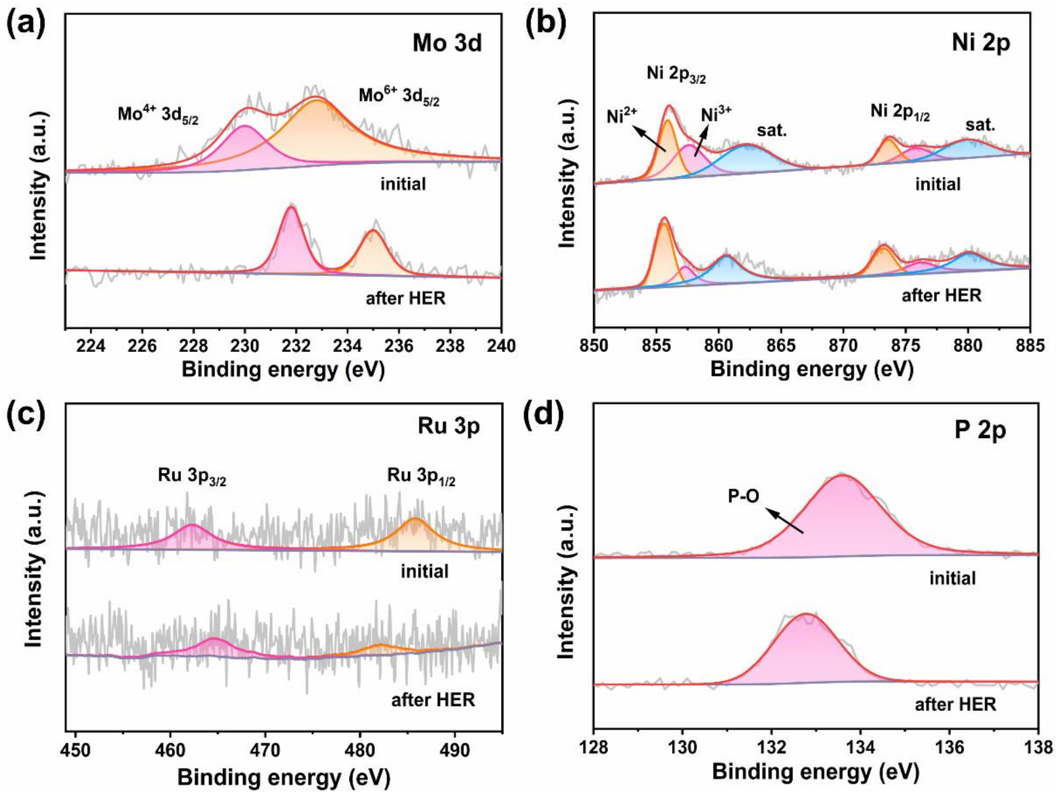

Figure S14. XPS spectra for a) Mo 3d, b) Ni 2p, c) Ru 3p, and d) P 2p of Ru-NiMoO(P)$_4$ before and after HER test.

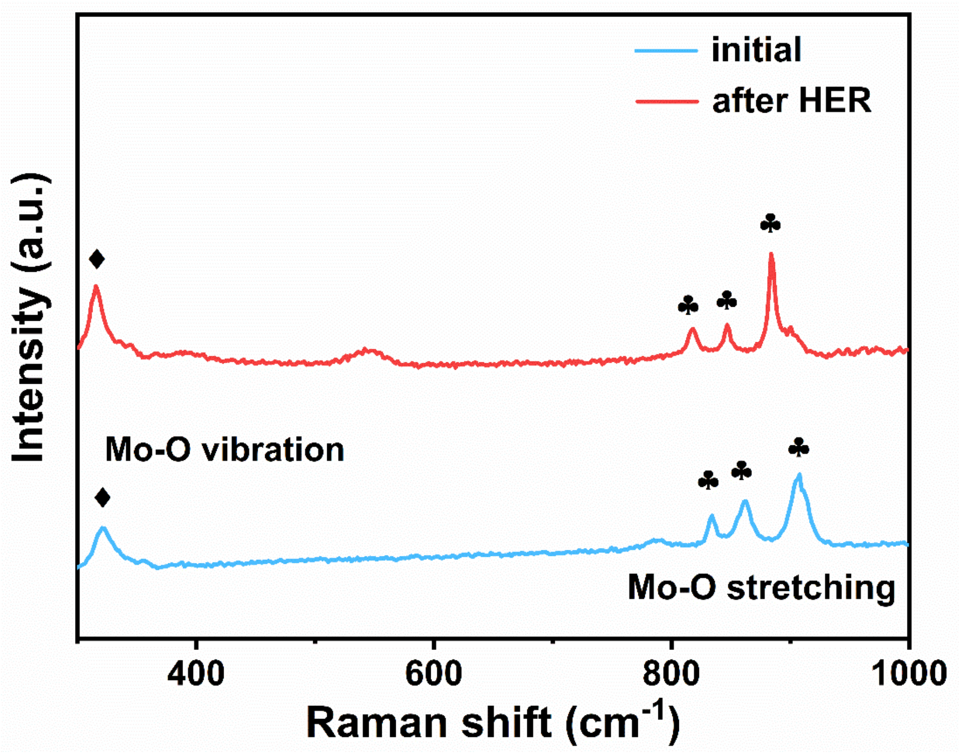

Figure S15. Raman spectra of Ru-NiMoO(P)$_4$ before and after HER test.

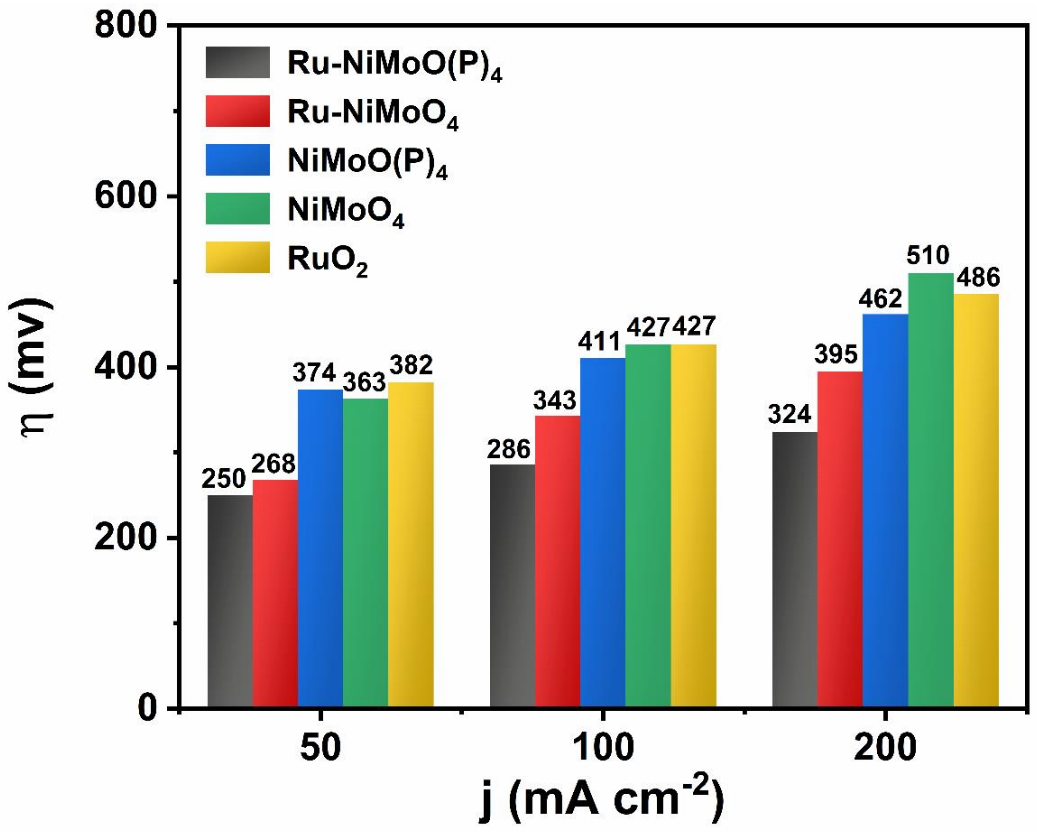

Figure S16. Corresponding overpotentials (@50, 100, 200 mA cm^{-2}) of OER performance for Ru-$NiMoO(P)_4$, Ru-$NiMoO_4$, $NiMoO(P)_4$, $NiMoO_4$ and commercial RuO_2 tested in 1 M KOH seawater.

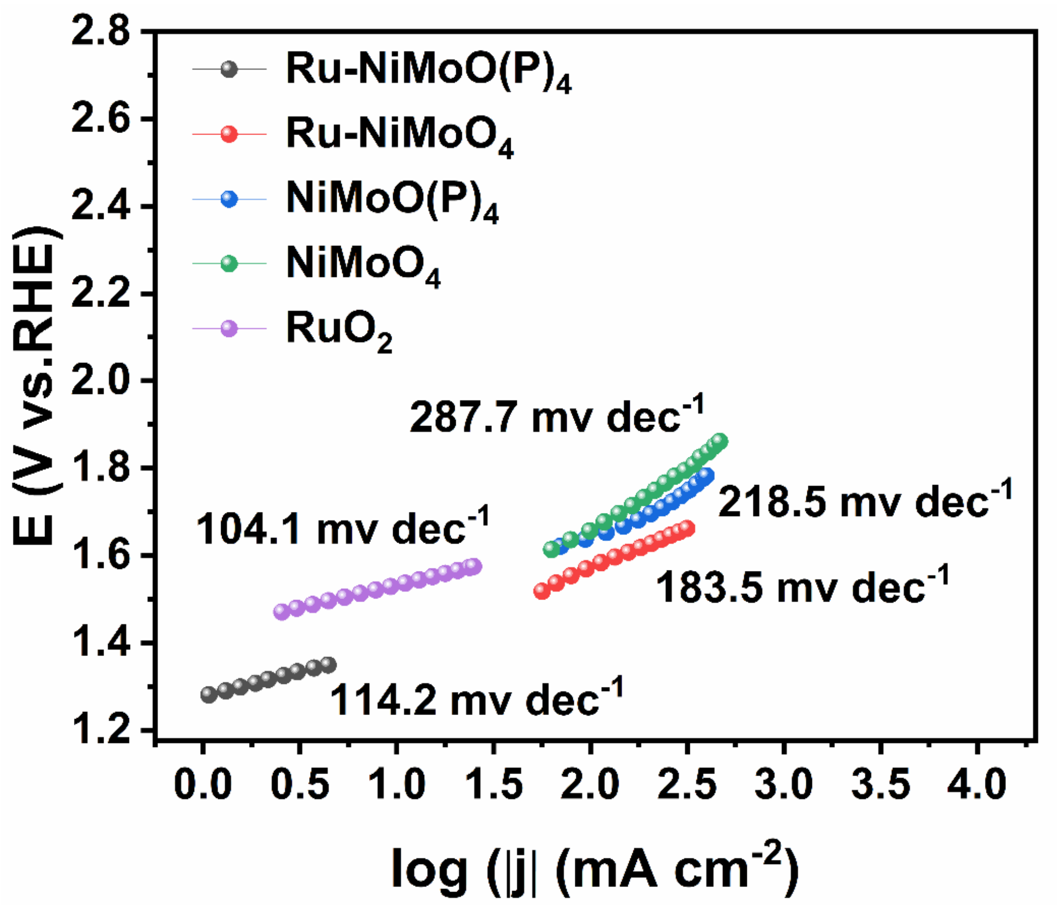

Figure S17. Tafel slopes of OER performance for Ru-$NiMoO(P)_4$, Ru-$NiMoO_4$, $NiMoO(P)_4$, $NiMoO_4$ and commercial RuO_2 tested in 1 M KOH seawater.

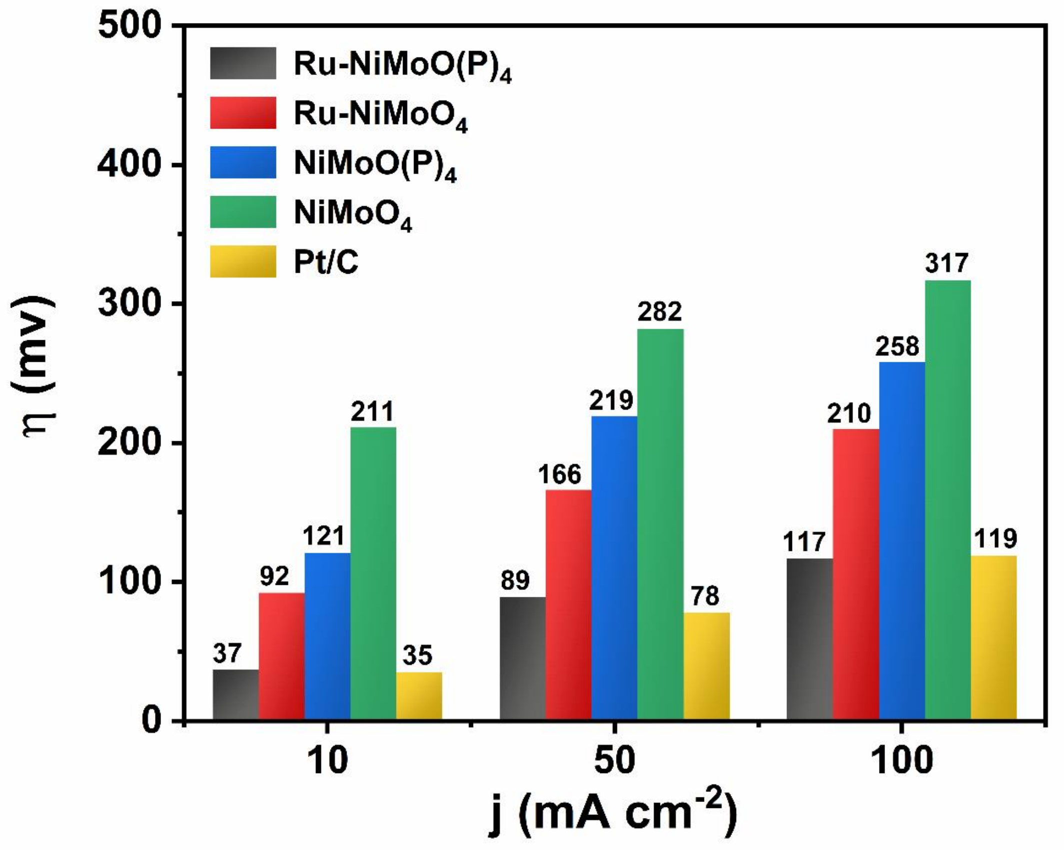

Figure S18. Corresponding overpotentials (@10, 50, 100 mA cm^{-2}) of HER performance for Ru-$NiMoO(P)_4$, Ru-$NiMoO_4$, $NiMoO(P)_4$, $NiMoO_4$ and commercial Pt/C tested in 1 M KOH seawater.

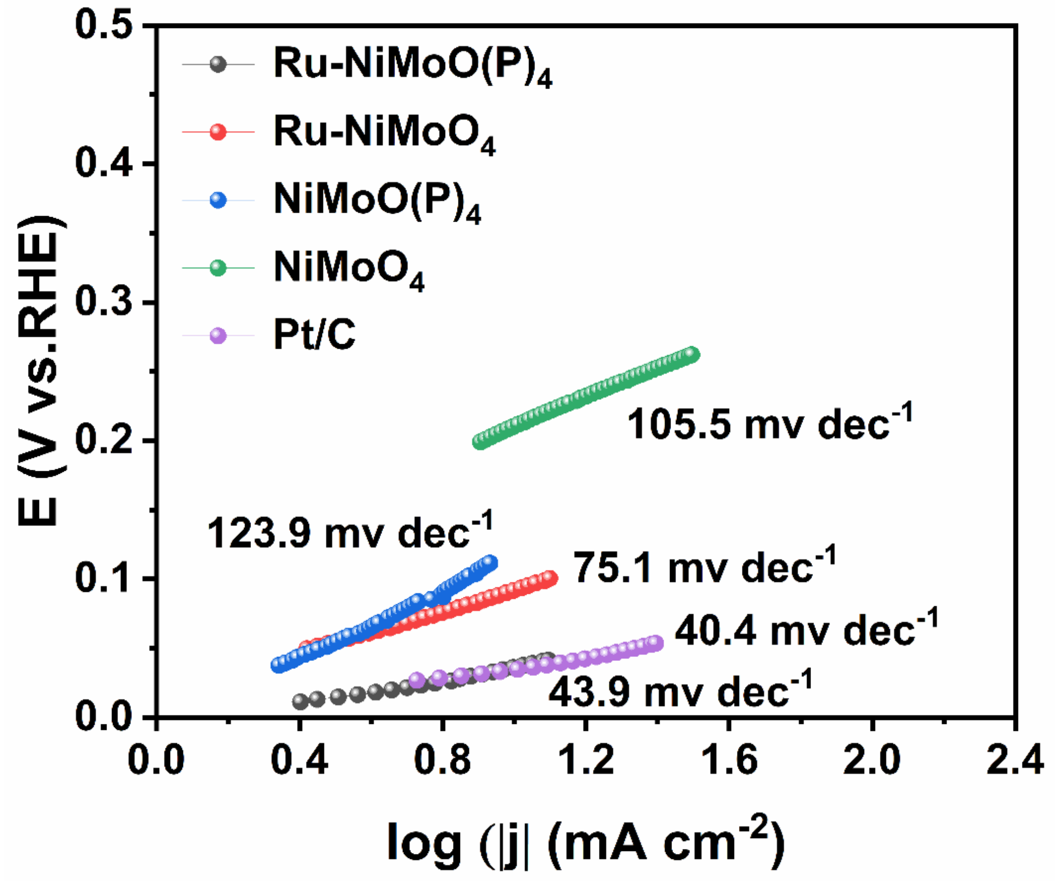

Figure S19. Tafel slopes of OER performance for Ru-$NiMoO(P)_4$, Ru-$NiMoO_4$, $NiMoO(P)_4$, $NiMoO_4$ and commercial Pt/C tested in 1 M KOH seawater.

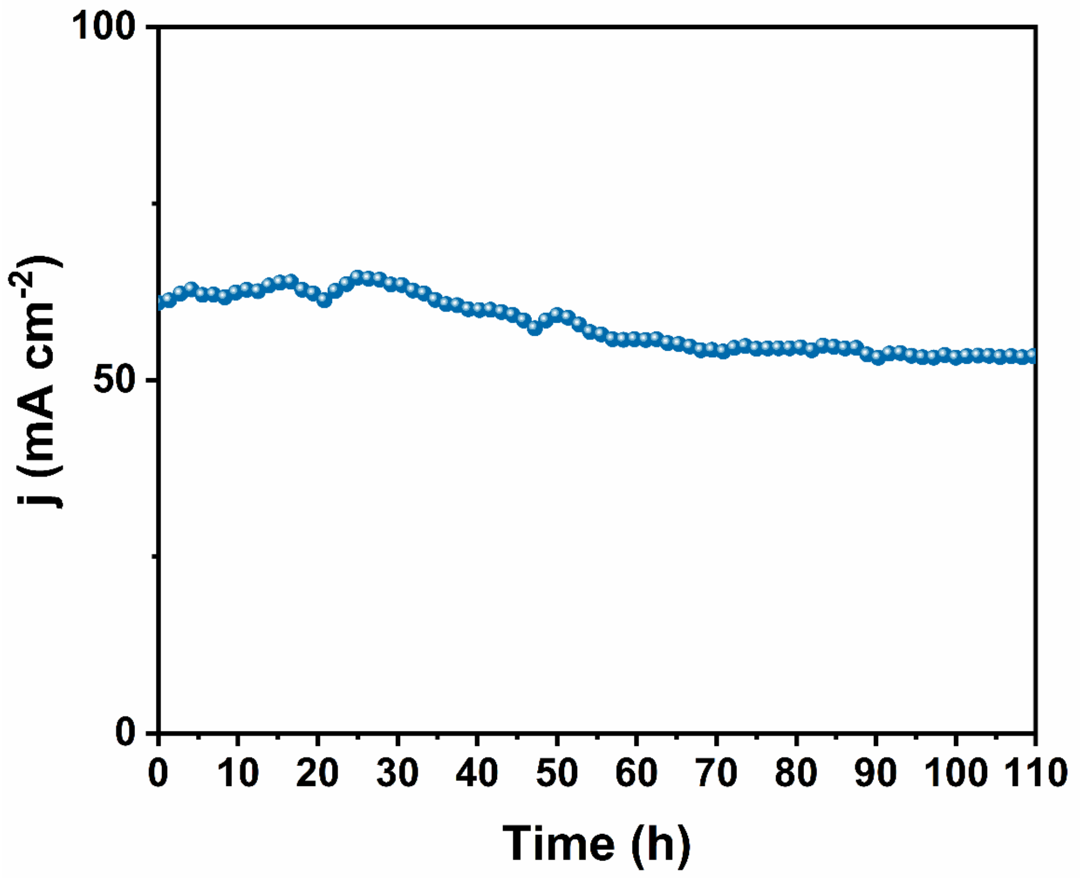

Figure S20. Chronopotentiometric curve of OER performance for Ru-NiMoO(P)$_4$ in 1 M KOH seawater.

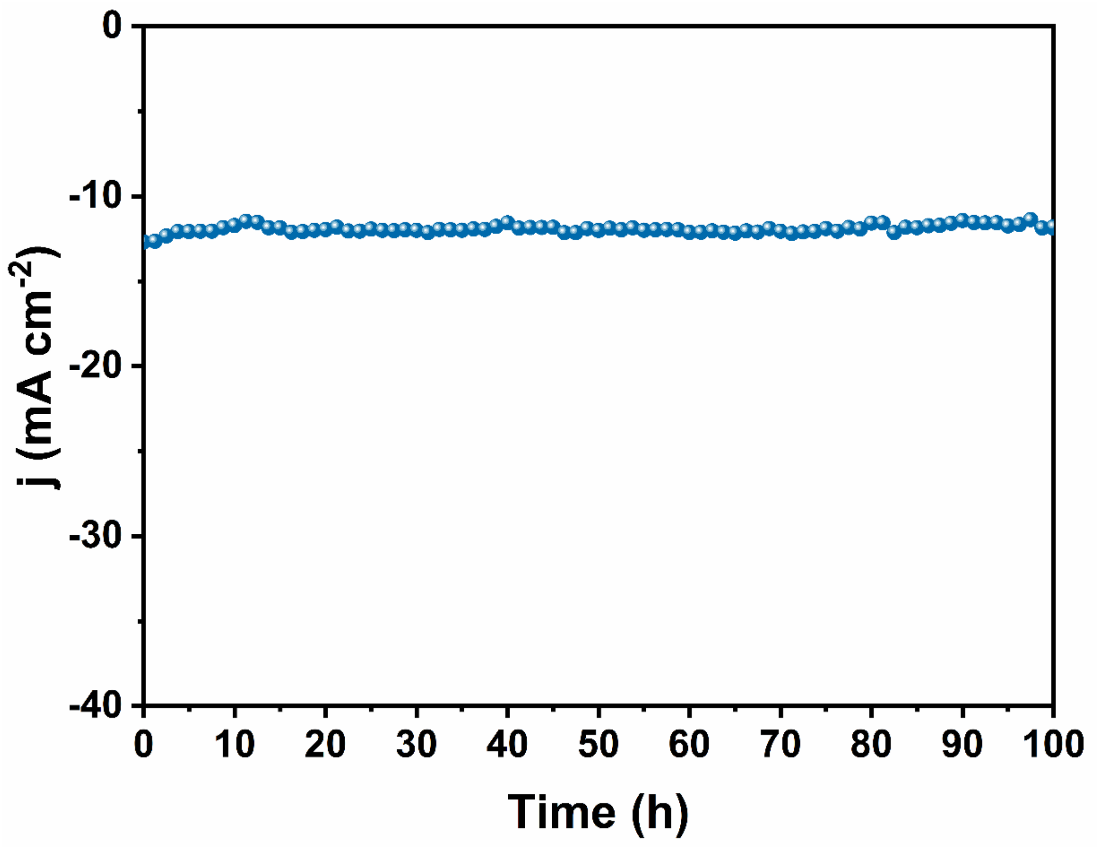

Figure S21. Chronopotentiometric curve of HER performance for Ru-NiMoO(P)$_4$ in 1 M KOH seawater.

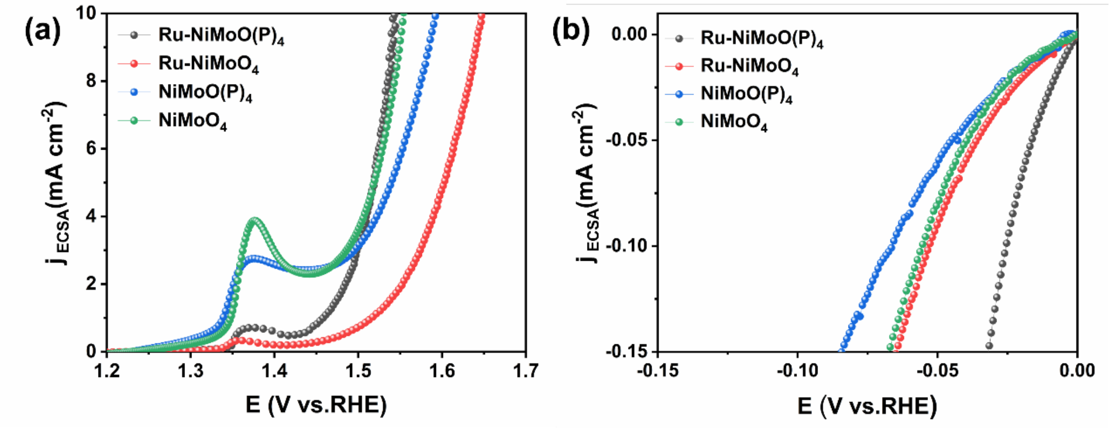

Figure S22. ECSA normalized polarization curves of the samples electrodes toward a) OER and b) HER.

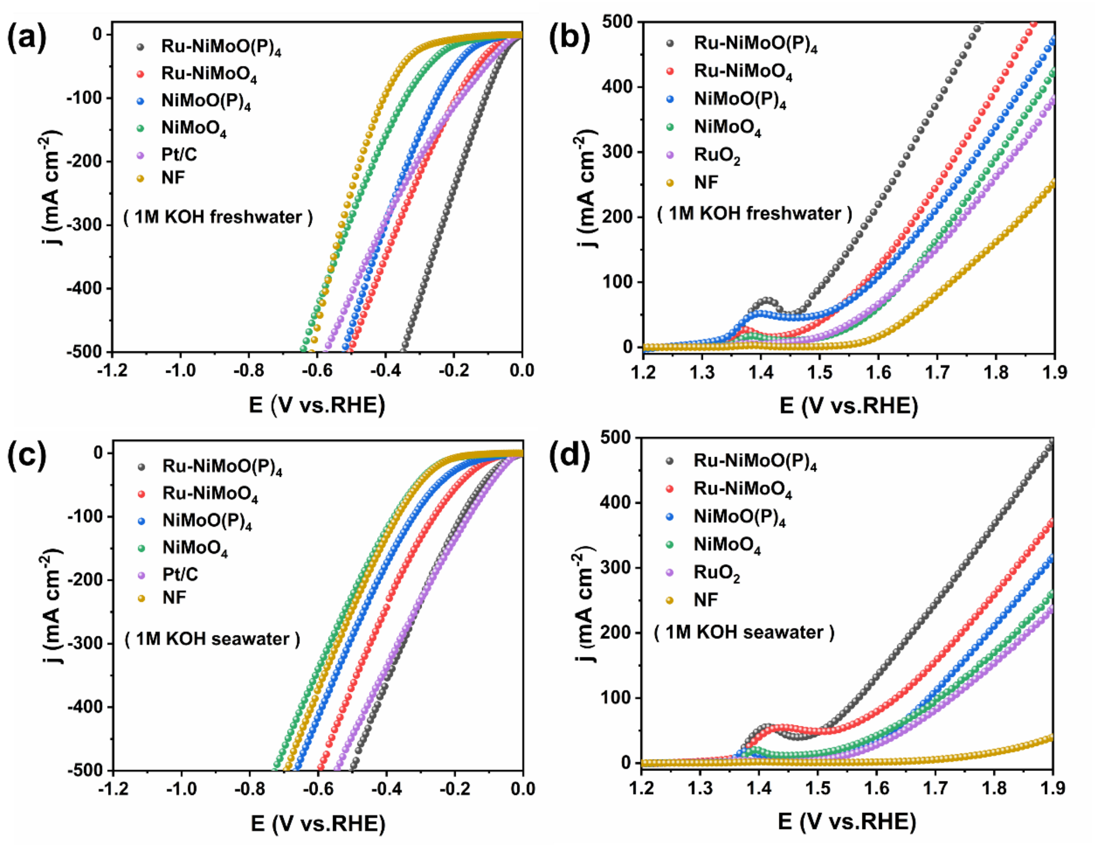

Figure S23. LSV curves of the samples for a) the HER in alkaline freshwater without iR corrections, b) the OER in alkaline freshwater without iR corrections, c) the HER in alkaline seawater media without iR corrections and d) the OER in alkaline seawater media without iR corrections.

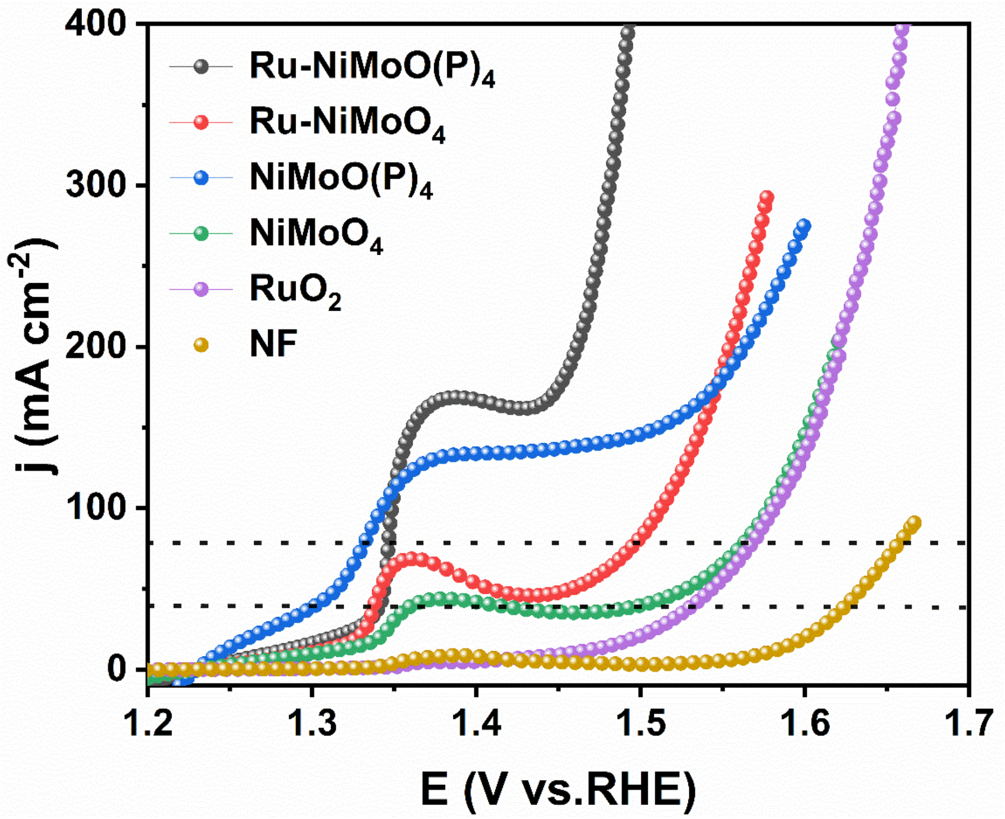

Figure S24. Polarization curves of Ru-NiMoO(P)$_4$/NF for the OER continuous potential sweeps at 5 mV·s^{-1} in alkaline media.

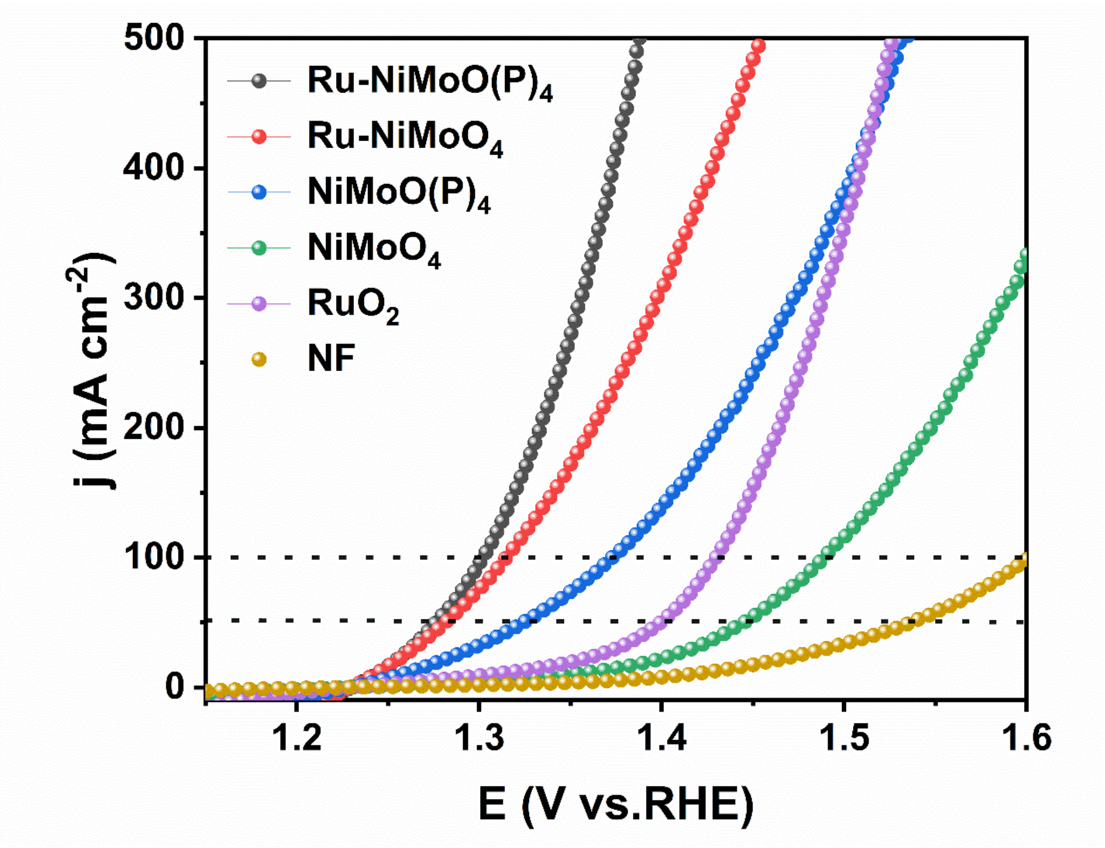

Figure S25. Polarization curves of Ru-NiMoO(P)$_4$/NF for the OER continuous reverse sweeps in alkaline media.

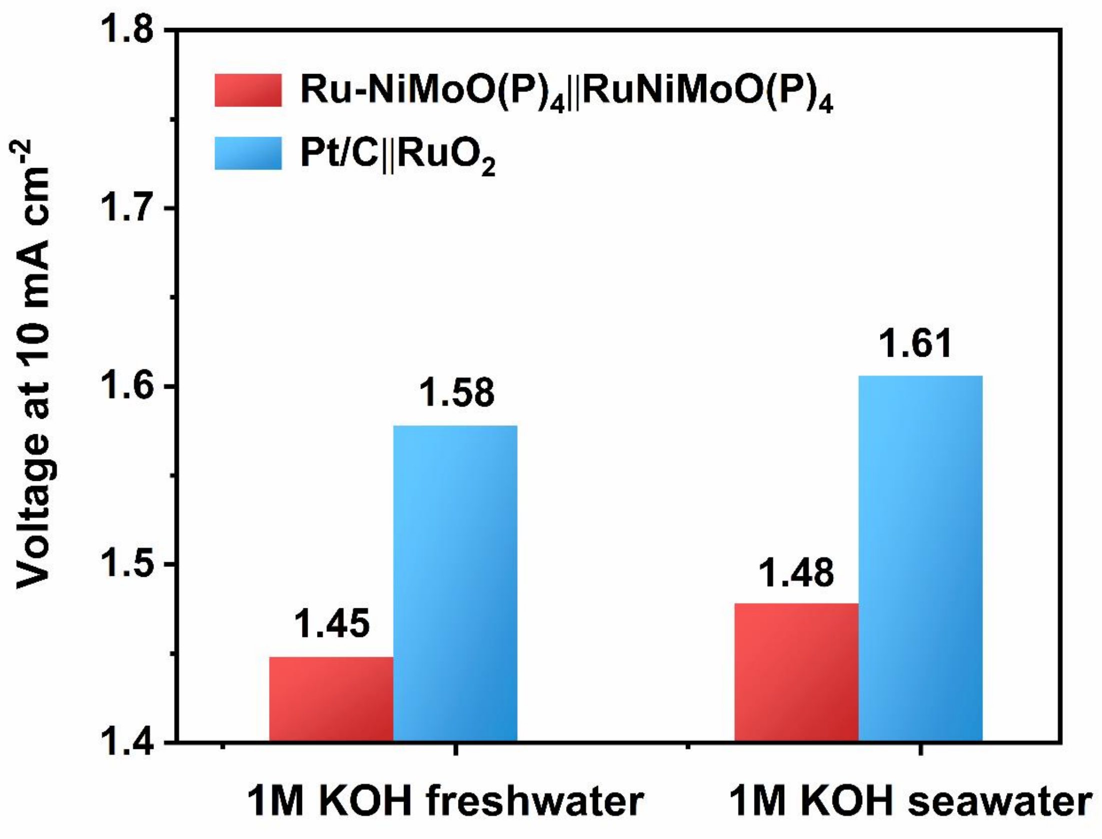

Figure S26. Corresponding cell voltages of Ru-NiMoO(P)$_4$||Ru-NiMoO(P)$_4$ and Pt/C||RuO$_2$ for water splitting in 1 M KOH freshwater and 1 M KOH seawater. (non-iR-correction).

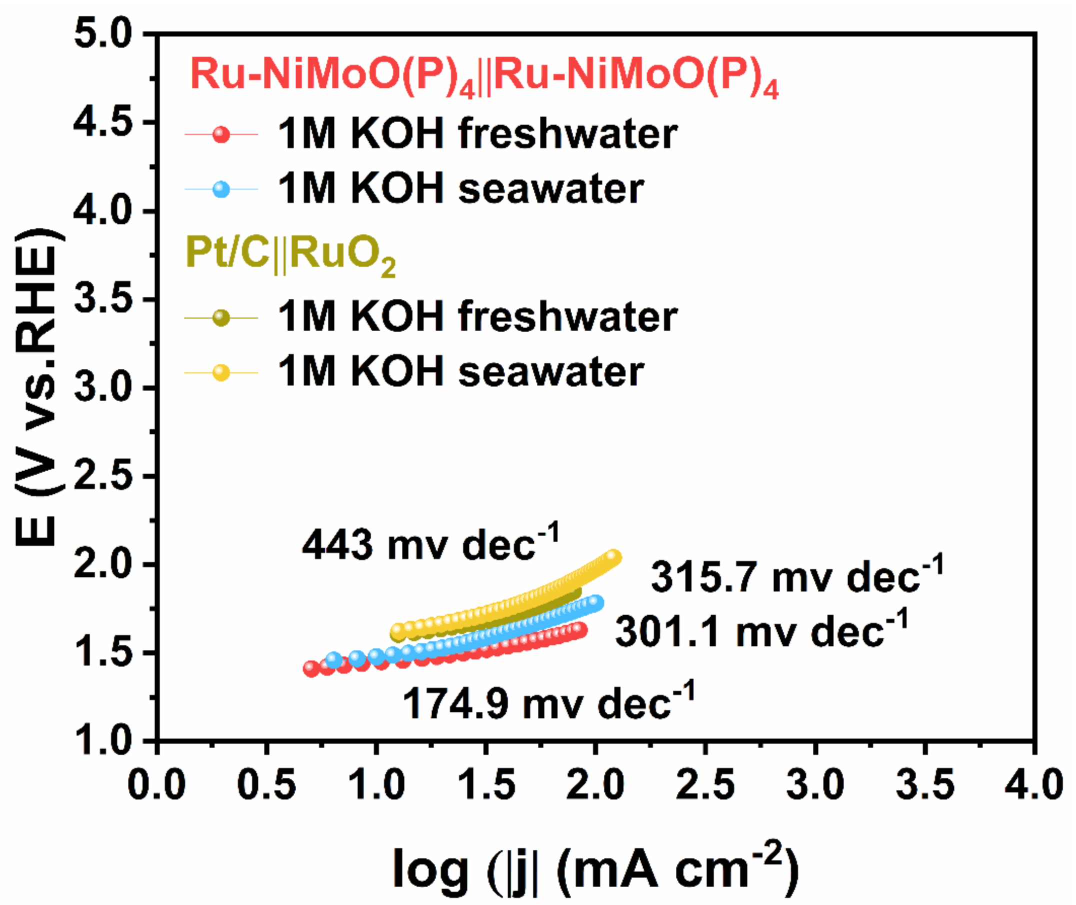

Figure S27. Tafel plots of Ru-NiMoO(P)$_4$||Ru-NiMoO(P)$_4$ and Pt/C||RuO_2 for water splitting in 1 M KOH freshwater and 1 M KOH seawater.

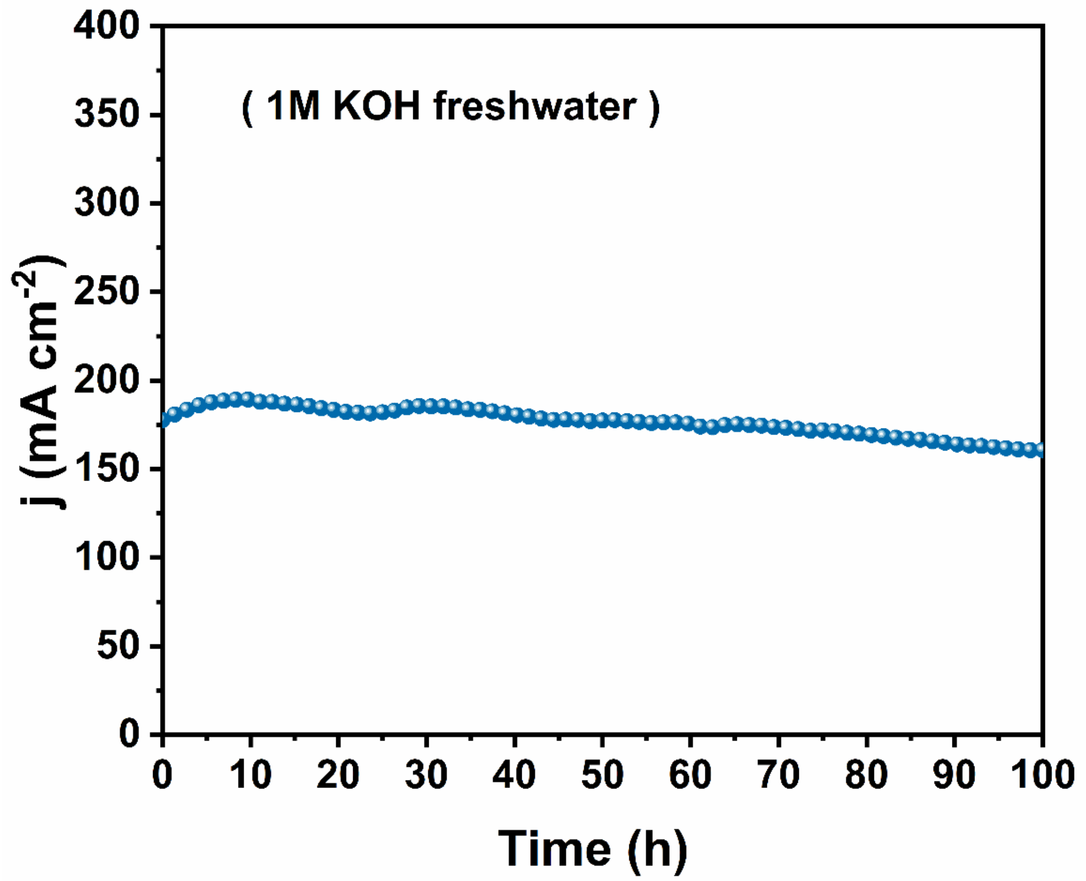

Figure S28. Chronopotentiometric curve of Ru-NiMoO(P)$_4$||Ru-NiMoO(P)$_4$ for water splitting in 1 M KOH freshwater.

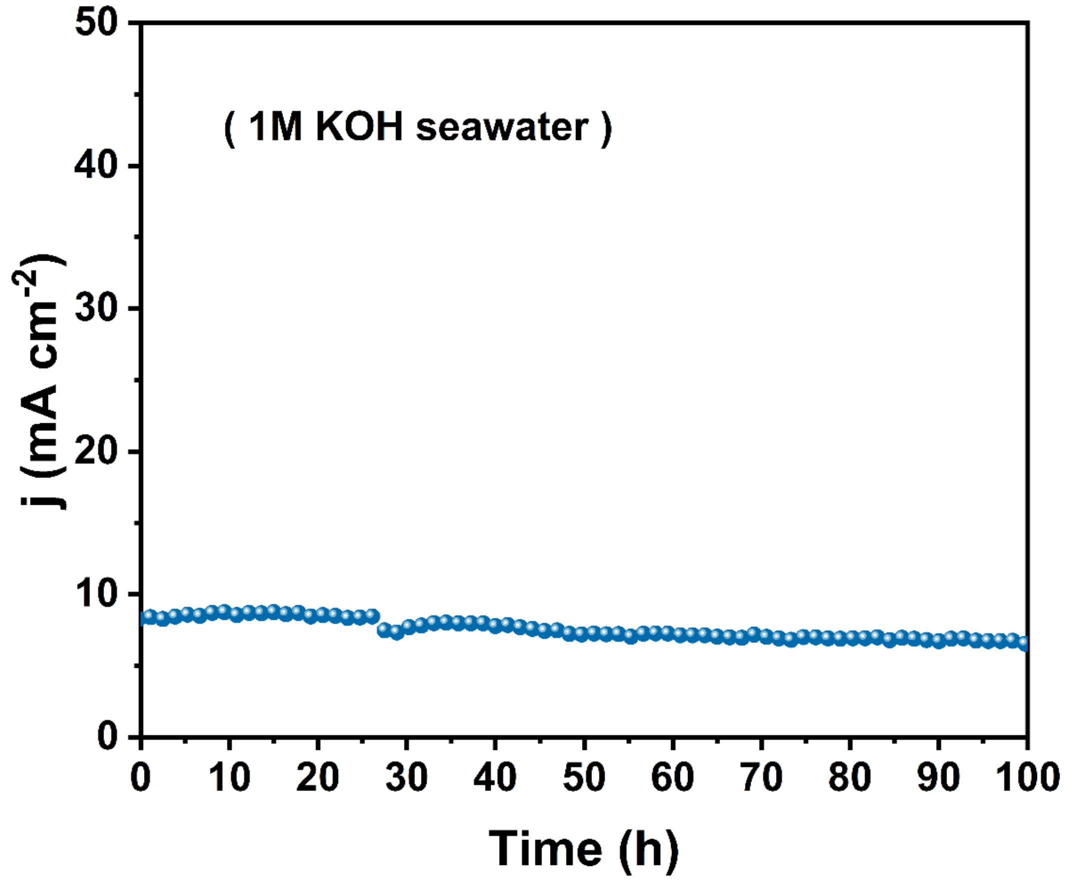

Figure S29. Chronopotentiometric curve for Ru-NiMoO(P)$_4$||Ru-NiMoO(P)$_4$ for water splitting in 1 M KOH seawater.

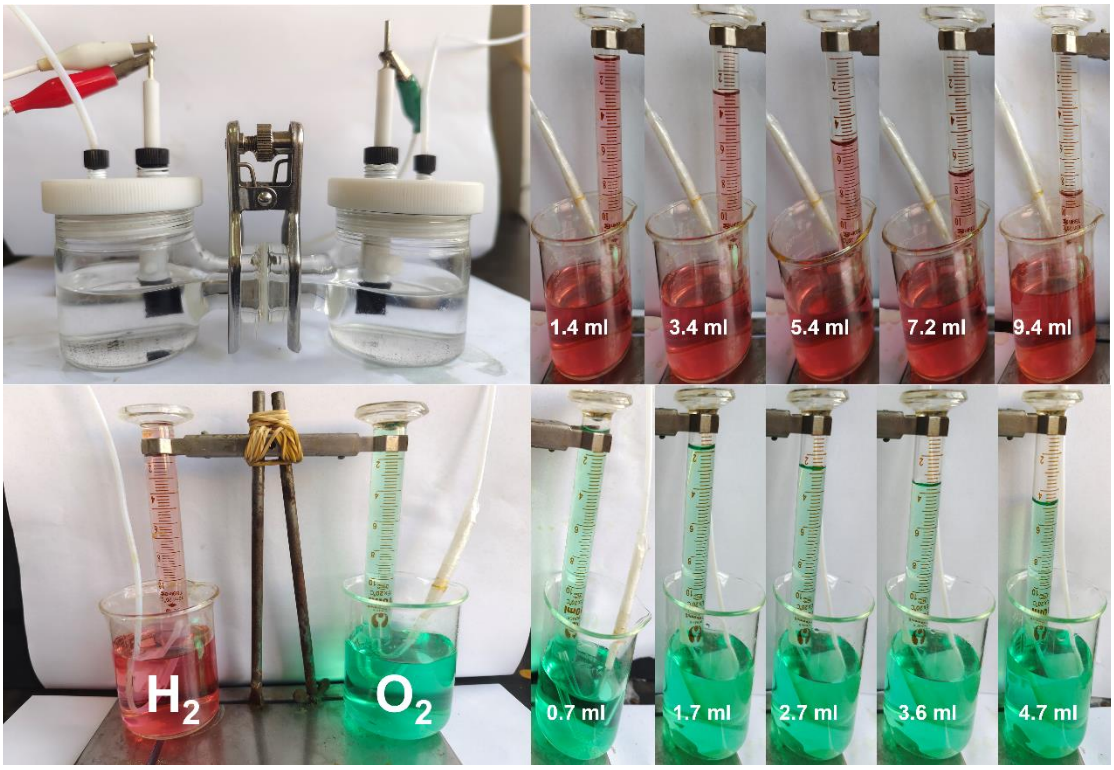

Figure S30. Digital photographs and volume of H_2 and O_2 experimentally measured at different time for Ru-NiMoO(P)$_4$ in 1M KOH freshwater.

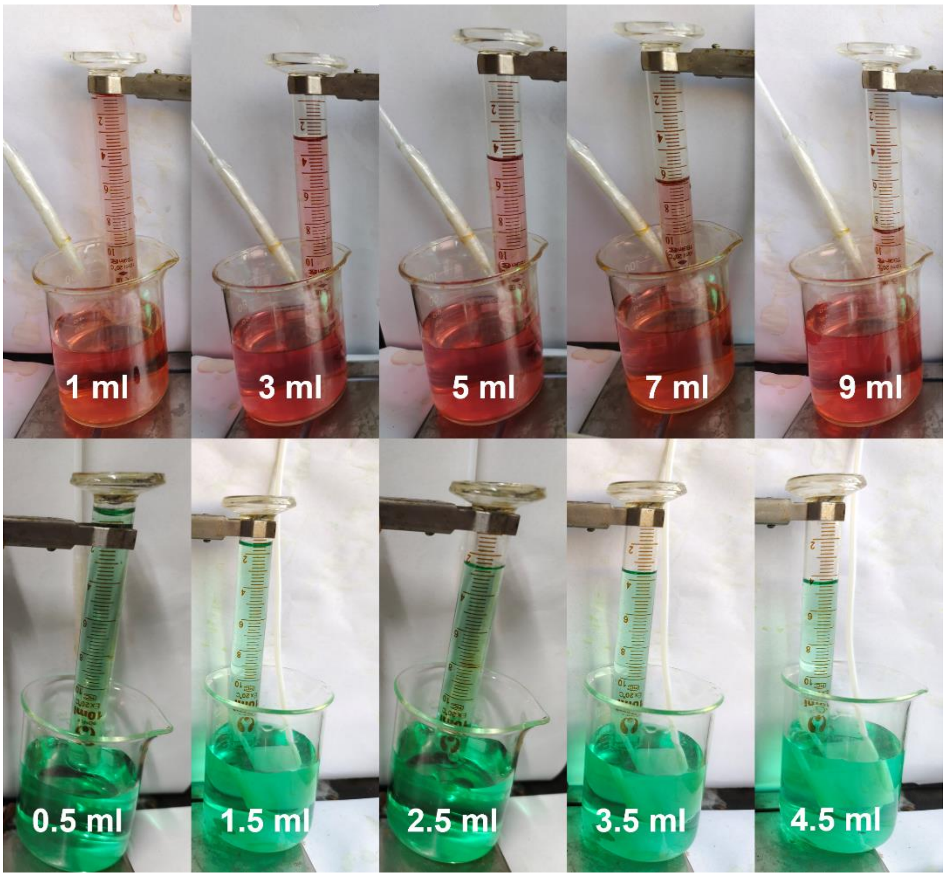

Figure S31. Digital photographs and volume of H_2 and O_2 experimentally measured at different time for Ru-NiMoO(P)$_4$ in 1M KOH seawater.

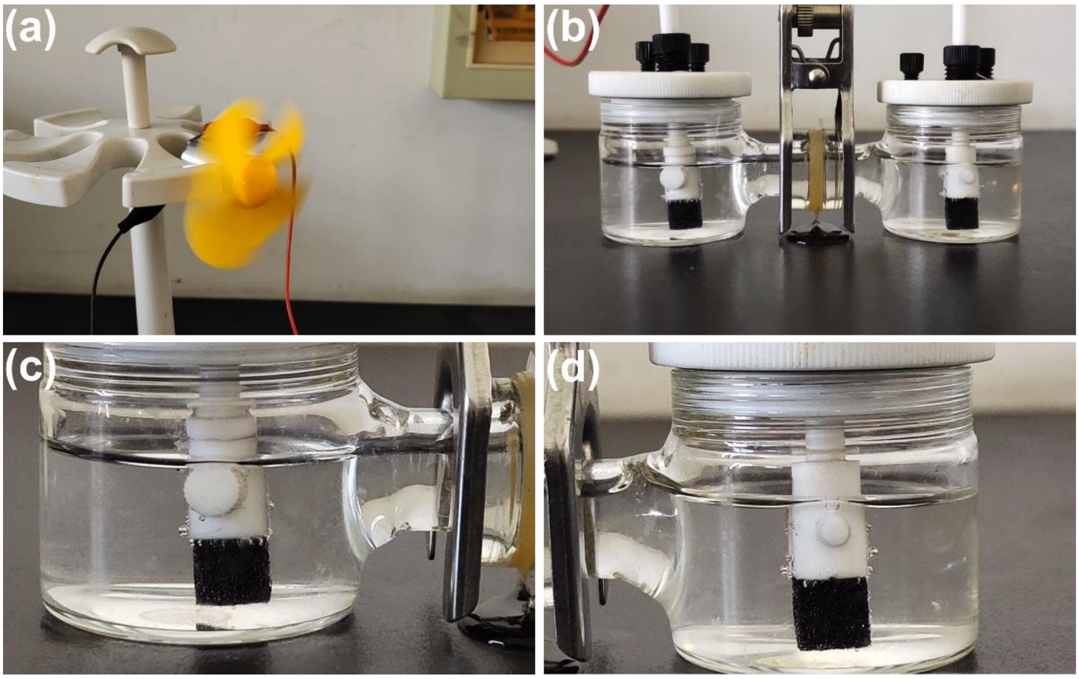

Figure S32. Digital photograph of the integrated wind-to-hydrogen system.

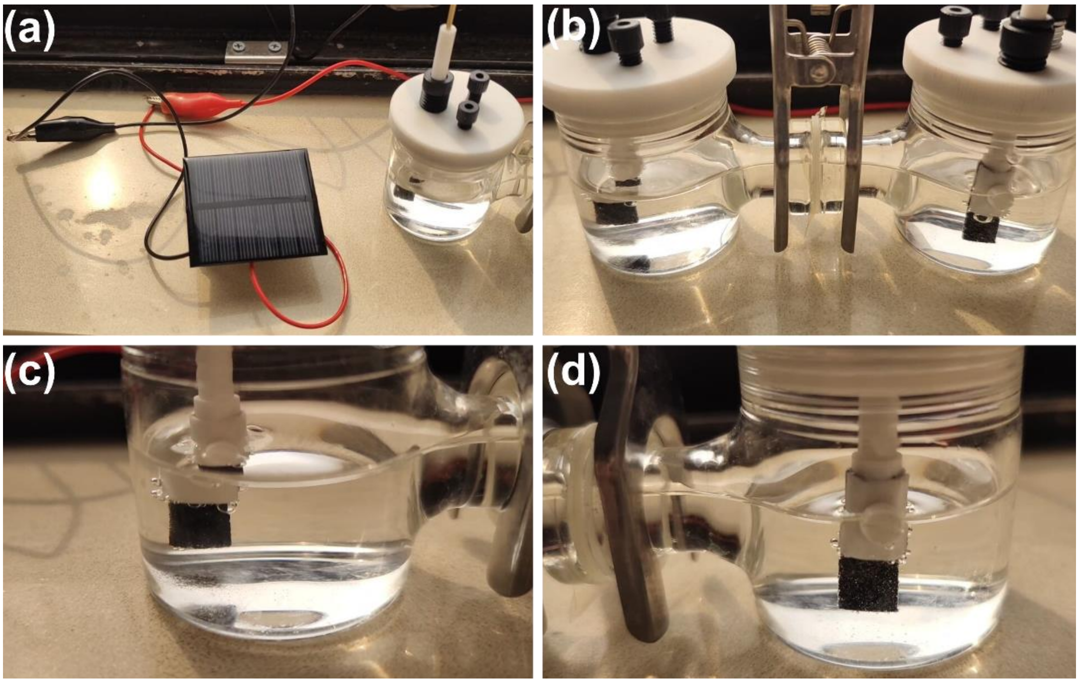

Figure S33. Digital photograph of the integrated solar-to-hydrogen system.

Table S1. The value of C_{dl} and corresponding ECSA for Ru-NiMoO(P)$_4$/NF, Ru-NiMoO$_4$/NF, NiMoO(P)$_4$/NF and NiMoO$_4$/NF in the region of 0.1 - 0.2 V versus Hg/HgO.

**Catalyst**	**Support**	**C_{dl} (mF cm^{-2})**	**ECSA (cm^2)**
Ru-NiMoO(P)$_4$	NF	203.71	101.86
Ru-NiMoO$_4$	NF	161.29	80.65
NiMoO(P)$_4$	NF	37.62	18.81
NiMoO$_4$	NF	9.05	4.53

Table S2. The value of C_{dl} and corresponding ECSA for Ru-$NiMoO(P)_4$/NF, Ru-$NiMoO_4$/NF, $NiMoO(P)_4$/NF and $NiMoO_4$/NF in the region of (-0.87) - (-0.77) V versus Hg/HgO.

**Catalyst**	**Support**	**C_{dl} (mF cm^{-2})**	**ECSA (cm^2)**
Ru-$NiMoO(P)_4$	NF	231.88	115.94
Ru-$NiMoO_4$	NF	204.38	102.19
$NiMoO(P)_4$	NF	89.98	44.99
$NiMoO_4$	NF	25.03	12.52

Table S3. Comparison of OER performance of Ru-NiMoO(P)$_4$/NF with other reported highly active OER electrocatalysts in 1 M KOH freshwater.

**Catalyst**	**Overpotential @ j (mV @ mA cm^{-2})**	**Ref.**
Ru-NiMoO(P)$_4$	194@50	This work
Ru-NiMoO(P)$_4$	265@100	This work
Ru-NiMoO(P)$_4$	285@200	This work
$Ni_{0.85}Se$-O/CN	240@10	1
CoNi-BDC@LDH	282@100	2
NiCoFe-HO@NiCo-LDH	278@10	3
NiFeW LDH	248@20	4
Ru-NiO/Co_3O_4	269@100	5
Ni-BDC-1	225@10	6
Ru/Co-N-C	232@10	7
RuO_X NCs	266@10	8
Ru/Co_3O_{4-x}	280@10	9
Ru-CoP/NC	330@10	10

Table S4. Comparison of HER performance of Ru-NiMoO(P)$_4$/NF with other reported active HER electrocatalysts in 1 M KOH.

**Catalyst**	**Overpotential @ j (mV @ mA cm^{-2})**	**Ref.**
Ru-NiMoO(P)$_4$	24@10	This work
Commercial Pt/C	32@10	This work
Ru@Ni-MOFs	25@10	11
CoNiP/MP Ni	37@10	12
α-Co(OH)$_2$@Ru	33@10	13
CoRu-BPDC	37@10	14
Ru-NiFe-P	44@10	15
CeO$_2$-NiCoP$_x$/NCF	39@10	16
Ru/Co$_3$O$_4$ NWs	30.96@10	17
Ru-MoS$_2$@PPy	37@10	18
Ru/Ni-MoS$_2$	32@10	19
a-Ru@Co-DHC	40@10	20

Table S5. Comparison of water splitting performance of Ru-NiMoO(P)$_4$/NF with other reported highly active bifunctional electrocatalysts in 1 M KOH.

**Catalyst**	**Voltage @ j (mV @ mA cm^{-2})**	**Ref.**
Ru-NiMoO(P)$_4$	1.45@10	This work
Pt/C || RuO_2	1.58@10	This work
Ru/NiFe(OH)$_x$/NiFe-MOF || Pt/C	1.54@10	21
RuV-CoNiP	1.469@10	22
Ru-NiSe$_2$	1.537@10	23
Ru@MoO(S)$_3$	1.522@20	24
Ru-NiCoP	1.515@10	25
Ni_2P/Ni_3S_2	1.50@10	26
NiFe-LDH@CoS$_x$	1.537@10	27
Pt-Co-Mo	1.50 V@10	28
SiO$_x$/Ru NSs	1.496@10	29
Ru-NiFeP	1.47@10	30

Table S6. Comparison of OER and HER performance of Ru-NiMoO(P)$_4$/NF with other reported electrocatalysts in alkaline seawater (1 M KOH seawater).

**Catalyst**	**OER Overpotential @ j (mV @ mA cm^{-2})**	**HER Overpotential @ j (mV @ mA cm^{-2})**	**Ref.**
Ru-NiMoO(P)$_4$	250@50	37@10	This work
Ru SAs-MoO$_{3-x}$	230@10	43@10	31
Ni$_2$P-Fe$_2$P	305@100	252@100	32
Ni$_x$Fe$_y$N@C	283@100	142@100	33
NiFe/Fe-MoO$_2$	283@100	38@10	34
Ni$_2$P/NiS$_2$	344@100	188@100	35
S-NiMoO$_4$@NiFe-LDH	315@100	220@100	36
NiCoS	360@100	145@100	37
Co-Fe$_2$P	274@100	221@100	38
(NiFeCoV)S$_2$	299@100	—	39
Mo-NiS@NiTe	—	57@10	40

References

[1] Core-shell heterojunction engineering of $Ni_{0.85}Se$-O/CN electrocatalyst for efficient OER. https://doi.org/10.1016/j.cej.2022.140291

[2] Lattice oxygen-mediated Ni-O-O-M formation for efficient oxygen evolution reaction in MOF@LDH core-shell structures. https://doi.org/10.1016/j.cej.2022.140403

[3] Construction of Ni-Co-Fe Hydr(oxy)oxide@Ni-Co Layered Double Hydroxide Yolk-Shelled Microrods for Enhanced Oxygen Evolution. https://doi.org/10.1002/anie.202213049

[4] Stable and active NiFeW layered double hydroxide for enhanced electrocatalytic oxygen evolution reaction. https://doi.org/10.1016/j.cej.2021.130768

[5] Boosting the OER/ORR/HER activity of Ru-doped Ni/Co oxides heterostructure. https://doi.org/10.1016/j.cej.2022.135634

[6] Self-Reconstructed Metal-Organic Framework Heterojunction for Switchable Oxygen Evolution Reaction. https://doi.org/10.1002/anie.202214794

[7] Electronic Structure Engineering of Single-Atom Ru Sites via Co–N4 Sites for Bifunctional pH-Universal Water Splitting. https://doi.org/10.1002/adma.202110103

[8] One-pot pyrolysis synthesis of highly active Ru/RuO_x nanoclusters for water splitting. https://doi.org/10.1007/s12274-021-3590-x

[9] In Situ Immobilizing Atomically Dispersed Ru on Oxygen-Defective Co_3O_4 for Efficient Oxygen Evolution. https://pubs.acs.org/doi/10.1021/acscatal.2c04946

[10] Tuning the Electronic Structure of CoP Embedded in N-Doped Porous Carbon

Nanocubes Via Ru Doping for Efficient Hydrogen Evolution.
https://pubs.acs.org/doi/10.1021/acsami.1c14387

[11] The key role of carboxylate ligands in Ru@Ni-MOFs/NF in promoting water dissociation kinetics for effective hydrogen evolution in alkaline media. https://doi.org/10.1016/j.cej.2022.138618

[12] In-Situ construction of hierarchically porous CoNiP/MP Ni electrocatalyst for overall water splitting. https://doi.org/10.1016/j.fuel.2023.128400

[13] Activating Ru-O-Co Interaction on the α-$Co(OH)_2$@Ru Interface for Accelerating the Volmer Step of Alkaline Hydrogen Evolution.
https://doi.org/10.1002/smtd.202201362

[14] Electronic Modulation of Metal-Organic Frameworks Caused by Atomically Dispersed Ru for Efficient Hydrogen Evolution.
https://doi.org/10.1002/smll.202301850

[15] Regulating electron density of NiFe-P nanosheets electrocatalysts by a trifle of Ru for high-efficient overall water splitting.
https://doi.org/10.1016/j.apcatb.2019.118324

[16] Multiphase nanosheet-nanowire cerium oxide and nickel-cobalt phosphide for highly-efficient electrocatalytic overall water splitting.
https://doi.org/10.1016/j.apcatb.2022.121678

[17] Charge redistribution of Ru nanoclusters on Co_3O_4 porous nanowire via the oxygen regulation for enhanced hydrogen evolution reaction.
https://doi.org/10.1016/j.nanoen.2021.105940

[18] Ru-MoS_2@PPy hollow nanowire as an ultra-stable catalyst for alkaline hydrogen evolution reaction. https://doi.org/10.1016/j.ijhydene.2022.08.300

[19] Dual-metallic single Ru and Ni atoms decoration of MoS_2 for high-efficiency hydrogen production. https://doi.org/10.1016/j.apcatb.2021.120557

[20] Amorphous Ru nanoclusters onto Co-doped 1D carbon nanocages enables efficient hydrogen evolution catalysis. https://doi.org/10.1016/S1872-2067(21)63921-9

[21] In situ etch engineering of Ru doped NiFe$(OH)_x$/NiFe-MOF nanocomposites for boosting the oxygen evolution reaction. https://doi.org/10.1039/D1TA06438J

[22] Ultralow Ru-assisted and vanadium-doped flower-like CoP/Ni_2P heterostructure for efficient water splitting in alkali and seawater. https://doi.org/10.1039/D1TA08699E

[23] Ru-Incorporated Nickel Diselenide Nanosheet Arrays with Accelerated Adsorption Kinetics toward Overall Water Splitting. https://doi.org/10.1002/smll.202105305

[24] Anion-modulated molybdenum oxide enclosed ruthenium nano-capsules with almost the same water splitting capability in acidic and alkaline media. https://doi.org/10.1016/j.nanoen.2022.107445

[25] Ru-doped 3D flower-like bimetallic phosphide with a climbing effect on overall water splitting. https://doi.org/10.1016/j.apcatb.2020.119396

[26] Three-dimensional-networked Ni_2P/Ni_3S_2 heteronanoflake arrays for highly enhanced electrochemical overall-water-splitting activity. https://doi.org/10.1016/j.nanoen.2018.06.048

[27] Self-supported NiFe-LDH@CoS_x nanosheet arrays grown on nickel foam as

efficient bifunctional electrocatalysts for overall water splitting.
https://doi.org/10.1016/j.cej.2021.129512

[28] Co-Mo microcolumns decorated with trace Pt for large current density hydrogen generation in alkaline seawater. https://doi.org/10.1016/j.apcatb.2022.121762

[29] Interface-Enhanced SiO_x/Ru Heterocatalysts for Efficient Electrochemical Water Splitting. https://pubs.acs.org/doi/10.1021/acsami.2c21953

[30] Ru doped bimetallic phosphide derived from 2D metal organic framework as active and robust electrocatalyst for water splitting.
https://doi.org/10.1016/j.apsusc.2020.147952

[31] Flower-Like Amorphous MoO_{3-x} Stabilized Ru Single Atoms for Efficient Overall Water/Seawater Splitting. https://doi.org/10.1002/advs.202300342

[32] Heterogeneous Bimetallic Phosphide Ni_2P-Fe_2P as an Efficient Bifunctional Catalyst for Water/Seawater Splitting. https://doi.org/10.1002/adfm.202006484

[33] Ni_xFe_yN@C microsheet arrays on Ni foam as an efficient and durable electrocatalyst for electrolytic splitting of alkaline seawater.
https://doi.org/10.1039/D1TA01292D

[34] Fe-incorporated Ni/MoO_2 Hollow Heterostructure Nanorod Arrays for High-efficiency Overall Water Splitting in Alkaline and Seawater Media.
https://doi.org/10.1002/smll.202205683

[35] Synergistically enhanced activity and stability of bifunctional nickel phosphide/sulfide heterointerface electrodes for direct alkaline seawater electrolysis.
https://doi.org/10.1016/j.jechem.2022.08.019

[36] Electrodeposition of NiFe-layered double hydroxide layer on sulfur-modified nickel molybdate nanorods for highly efficient seawater splitting. https://doi.org/10.1016/j.jcis.2022.01.044

[37] Heterogeneous bimetallic sulfides based seawater electrolysis towards stable industrial-level large current density. https://doi.org/10.1016/j.apcatb.2021.120071

[38] Synthesis of 3D heterostructure Co-doped Fe_2P electrocatalyst for overall seawater electrolysis. https://doi.org/10.1016/j.apcatb.2021.120386

[39] High-entropy NiFeCoV disulfides for enhanced alkaline water/seawater electrolysis. https://doi.org/10.1016/j.jcis.2023.04.172

[40] Robust and hydrophilic Mo-NiS@NiTe core-shell heterostructure nanorod arrays for efficient hydrogen evolution reaction in alkaline freshwater and seawater. https://doi.org/10.1016/j.apsusc.2023.157977
